# Effects of 1*H*-1,2,3-Triazole Derivatives of 3-*O*-Acetyl-11-Keto-Beta-Boswellic Acid from *Boswellia sacra* Resin on T-Cell Proliferation and Activation

**DOI:** 10.3390/ph17121650

**Published:** 2024-12-08

**Authors:** Abdo Meyiah, Satya Kumar Avula, Ahmed Al-Harrasi, Eyad Elkord

**Affiliations:** 1 Department of Biosciences and Bioinformatics and Suzhou Municipal Key Laboratory of Biomedical Sciences and Translational Immunology, School of Science, Xi’an Jiaotong-Liverpool University, Suzhou 215123, China; abdo.meyiah@xjtlu.edu.cn; 2 Natural and Medical Sciences Research Center, University of Nizwa, Nizwa 616, Oman; chemisatya@gmail.com; 3 College of Health Sciences, Abu Dhabi University, Abu Dhabi 59911, United Arab Emirates; 4 Biomedical Research Center, School of Science, Engineering and Environment, University of Salford, Manchester M5 4WT, UK

**Keywords:** boswellic acid, T cells, CD25, *β*-AKBA, 1*H*-1,2,3-triazole

## Abstract

**Background:** 3-*O*-acetyl-11-keto-*β*-boswellic acid (*β*-AKBA), a triterpene natural product, is one of the main natural products of *Boswellia sacra* resin (BSR) and has reported biological and immunomodulatory effects. 1*H*-1,2,3-triazole derivatives of *β*-AKBA (named **6a**–**6d**) were synthesized from *β*-AKBA. The 1*H*-1,2,3-triazole compounds are also known to have a wide range of biological and pharmacological properties as demonstrated by in vitro and in vivo studies. This study aimed to investigate the effects of these 1*H*-1,2,3-triazole derivatives of *β*-AKBA on human T-cell proliferation and activation. **Methods:** PBMCs isolated from healthy donors were activated by anti-CD3/CD28 monoclonal antibodies in the presence of *β*-AKBA (**1**) or 1*H*-1,2,3-triazole derivatives of *β*-AKBA or DMSO controls. **Results:** We found that similar to the parent compound *β*-AKBA (**1**), derivatives **6a**, **6b**, and **6d** significantly inhibited T-cell expansion/proliferation and reduced the levels of CD25 activation marker on CD4^+^ and CD8^+^ T cells without exerting significant cytotoxic effects on T-cell viability at a concentration of 25 µM. However, compound **6c** further inhibited T-cell expansion/proliferation and CD25 expression, but had a significant cytotoxic effect on cell viability at similar concentrations of 25 µM. **Conclusions:** These findings demonstrate the immunoinhibitory effects of *β*-AKBA (**1**) and its corresponding triazole derivatives on T-cell proliferation and activation, highlighting the promising therapeutic potential of these compounds in T-cell-mediated diseases.

## 1. Introduction

*Boswellia sacra* resin (BSR) is a resin-producing tree that grows in South Oman (Dhofar region) [1], Yemen (Hadhramaut region), and northern Somalia [2]. BSR consists of various terpenes, which include mono- (C10), sesqui- (C15), di- (20), and tri-terpenes (C30). It is reported to possess antibacterial, anti-inflammatory, antidiabetic, and anticancer activities [3].

Boswellic acids (BAs) are natural products isolated from the resins of plants of the *boswellia* genus and are known for their medicinal potential [4]. The most popular BAs include *β*-boswellic acid (*β*-BA), 3-*O*-acetyl-*β*-boswellic acid (*β*-ABA), 11-keto-*β*-boswellic acid (*β*-KBA), and 3-*O*-acetyl-11-keto-*β*-boswellic acid (*β*-AKBA). Different BAs have shown promising therapeutic effects against various diseases such as inflammation-related disorders, including osteoarthritis, asthma, inflammatory bowel disease, and microbial infections, as well as Alzheimer’s disease [5,6,7,8,9,10]. In addition, BAs exhibit anticancer effects in a variety of tumors including breast, colorectal, lung, and gastric cancers [11,12,13,14].

The immunomodulatory activities of BAs were also demonstrated through their role in inhibiting proinflammatory cytokines and the classical complement pathway, as well as enhancing macrophage phagocytosis [15,16]. Furthermore, other studies reported that the immunomodulatory activity of BA mixtures is mediated by reducing the expression of T helper 1 (Th1) cytokines such as interferon-gamma (IFN-γ) and interleukin-2 (IL-2) and increasing the expression of Th2-related IL-4 and IL-10 following the treatment of mouse splenic T cells with BAs [17]. Proinflammatory cytokines derived from Th1 or Th17 cells are known to be involved in a wide range of biological processes, including inflammation and immune responses. For example, *β*-AKBA treatment reduces the differentiation of Th17 cells, which are required in the pathogenesis of autoimmune diseases, by inhibiting the IL-1*β*-mediated inhibition of IL-1 receptor-associated kinase 1 (IRAK1) [18]. Therefore, these natural products are believed to have immunomodulatory properties because they play dual roles in controlling cytokine production. On the other hand, the combination of *β*-AKBA with conventional therapeutics has been reported in different models through its role in enhancing anti-inflammatory and anticancer effects [19,20]. Therefore, reducing these cells can decrease the production of proinflammatory cytokines.

The 1*H*-1,2,3-triazole moiety is one of the nitrogen-heterocyclic compounds present in a variety of drugs. A benefit of 1*H*-1,2,3-triazole molecules is that they are stable for metabolic degradation. The presence of hydrogen bonding is ideal for binding with biomolecular targets and thereby improving their solubility [21]. In biomedical research, 1*H*-1,2,3-triazole possesses many potential pharmacological properties, including anticancer, anti-inflammatory, antidiabetic, antifungal, antiviral, and antibacterial activities [22,23,24,25,26,27,28,29,30,31].

In this study, a series of analogs of 1*H*-1,2,3-triazole derivatives of *β*-AKBA (**2**–**4** and **6a**–**6d**) from BSR were synthesized using a highly efficient “click chemistry reaction” technique. Although 1*H*-1,2,3-triazole derivatives are promising for treating several diseases, the immunomodulatory potential of *β*-AKBA-tethered 1*H*-1,2,3-triazole derivatives is not known. Therefore, more research is required to investigate their therapeutic effects. Our previous study showed that *β*-AKBA inhibited CD4^+^ and CD8^+^ T-cell proliferation and activation [32]. Herein, we used 1*H*-1,2,3-triazole derivatives of *β*-AKBA to investigate the therapeutic potential of these derivatives on immunomodulation by investigating their impact on the activation and proliferation of T cells.

## 2. Results

### 2.1. Effect of 1H-1,2,3-Triazole Derivatives of β-AKBA on T-Cell Expansion

**Figure 1:**
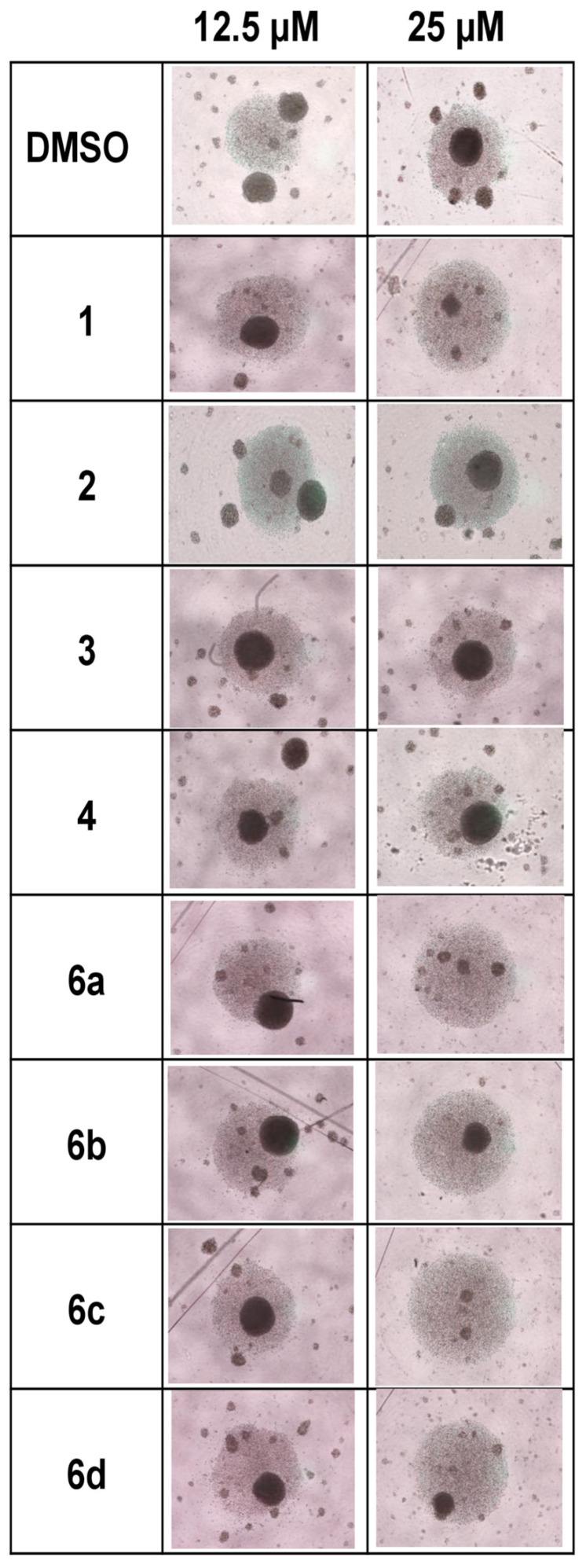
Peripheral blood mononuclear cells (PBMCs) were treated with the compounds or DMSO as control. Representative photomicrographs of T lymphocytes stimulated with anti-human CD3/CD28 antibodies and treated with two concentrations (12.5 and 25 µM) of *β*-AKBA **1**, **2**, **3**, **4**, **6a**, **6b**, **6c**, or **6d** derivatives as observed by microscopy after three days of stimulation.

We investigated the effect of compound **1** (β-AKBA) and its 1H-1,2,3-triazole derivatives, which included compounds **2**, **3**, **4**, and **6a**–**6d**, on activated T lymphocytes. Following 3 days of culture, the expansion of cells treated with DMSO controls, compound **1** (β-AKBA), and 1H-1,2,3-triazole derivatives of β-AKBA (**2**, **3**, **4** and **6a**–**6d**) was examined under a microscope. The microscopy images provided primary evidence that the synthesized compounds exerted inhibitory effects against T-cell expansion (Figure 1). Compared with DMSO control, compounds **1**, **6a**, **6b**, **6c**, and 6d reduced T-cell expansion at a concentration of 25 µM, but not at 12.5 µM, whereas compounds **2**, **3**, and **4** did not show any inhibitory effects at either concentration (Figure 1).

### 2.2. Effect of 1H-1,2,3-Triazole Derivatives of β-AKBA on T-Cell Viability

**Figure 2:**
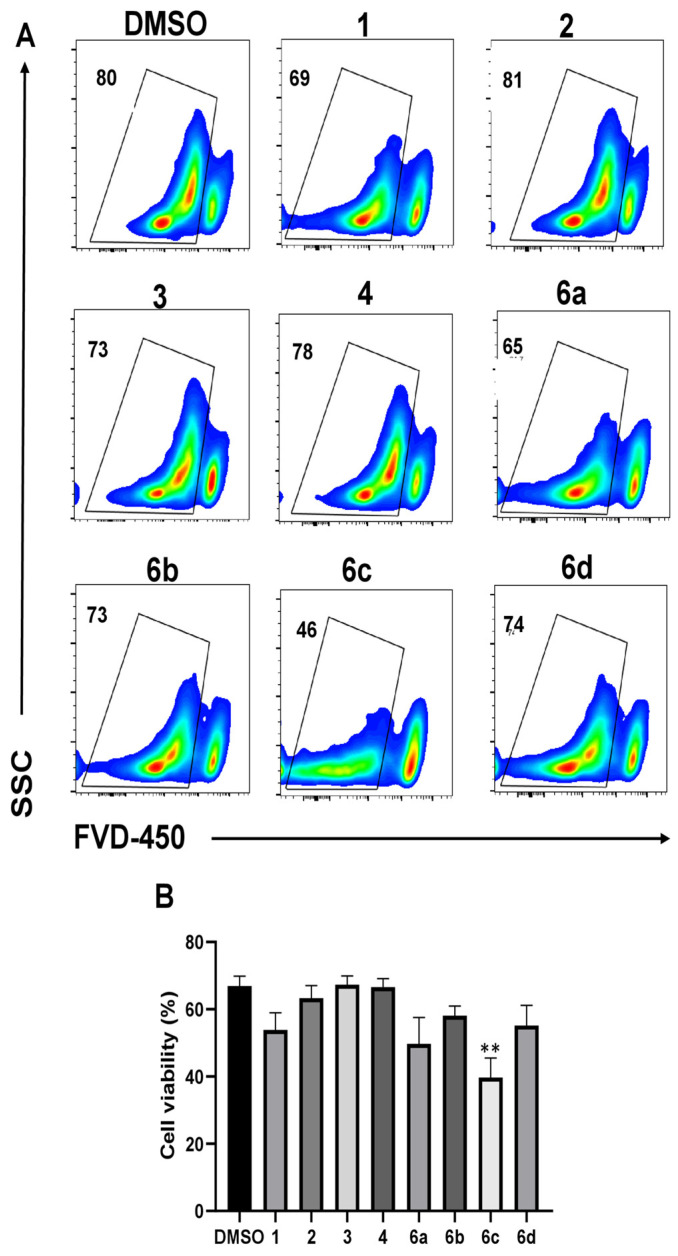
The effect of *β*-AKBA and derivative compounds at a concentration of 25 µM on T-cell viability after three days of stimulation was determined by FVD-450 dye and flow cytometry. Representative flow cytometric plots are shown in (**A**); gates indicate the percentage of viable cells. The overall viability bar plots for all experiments performed (n = 7) are shown in (**B**). Compound **6c** significantly reduced T-cell viability compared with DMSO. ** *p* < 0.01.

The gating strategy applied to the flow cytometric plots to define different cell subsets is illustrated in Supplementary Figure S1. The effects of β-AKBA and 1H-1,2,3-triazole derivatives of β-AKBA on cell viability were assessed after three days of T-cell stimulation via flow cytometry with a viability dye (FVD-450). In comparison to DMSO, β-AKBA and other derivative compounds at a concentration of 12.5 µM had no cytotoxic effects on the viability of T cells (Supplementary Figure S2). At a higher concentration of 25 µM, T-cell viability was relatively decreased in the presence of compounds **1**, **6a**, **6b**, and **6d** but not significantly reduced (Figure 2). Only compound **6c** induced a significant reduction in T-cell viability at a higher concentration (Figure 2B). Compared with β-AKBA, compound **6c** reduced the viability of T cells, although the difference did not reach statistical significance (*p* value = 0.09) (Figure 2B).

### 2.3. Effect of 1H-1,2,3-Triazole Derivatives of β-AKBA on T-Cell Proliferation

**Figure 3:**
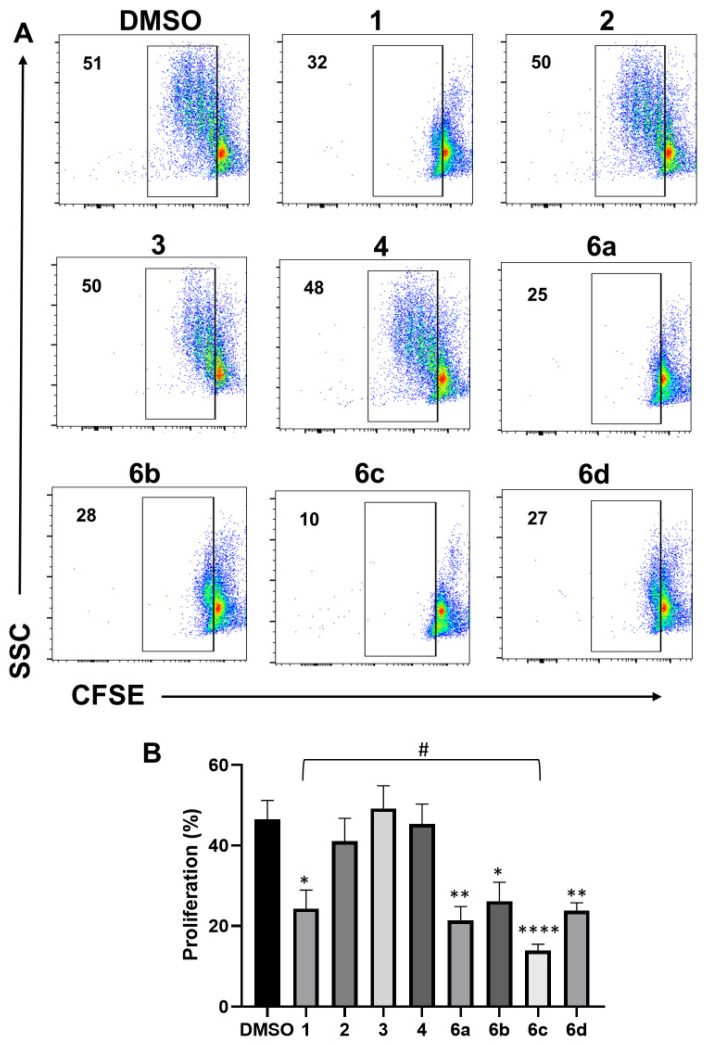
The effect of *β*-AKBA and derivative compounds at a 25 µM concentration on T-cell proliferation was determined by CFSE loss. Representative flow cytometric plots are shown in (**A**); gates indicate the percentage of proliferating cells The overall cell proliferation bar plots for all experiments performed (n = 8) are shown in (**B**). Compared with DMSO, the other compounds (**1**, **6a**, **6b**, **6c**, and **6d**) significantly reduced T-cell proliferation. Additionally, using an unpaired *t*-test, only compound **6c** significantly reduced T-cell proliferation compared to *β*-AKBA (*t*-test, # *p* < 0.05). * *p* < 0.05; ** *p* < 0.01; **** *p* < 0.0001).

Cell proliferation was evaluated after three days of culture by flow cytometric CFSE dilution assays. A positive-CFSE T-cell population indicates no proliferation, whereas a negative-CFSE T-cell population indicates proliferation. The percentages of cell proliferation remained unchanged when T cells were treated with β-AKBA or any of the other compounds at a 12.5 µM concentration (Supplementary Figure S3). Interestingly, compound **1** (β-AKBA) and compounds **6a**, **6b**, **6c**, and **6d** exhibited inhibitory effects at 25 µM by significantly reducing the percentage of T-cell proliferation, compared with the negative controls (DMSO) (Figure 3). Of note, the most significant reduction in T-cell proliferation was induced by compound **6c** (Figure 3B). Compared with β-AKBA, compound **6c** significantly reduced the proliferation of T cells (*p* value = 0.049).

### 2.4. Effect of 1H-1,2,3-Triazole Derivatives of β-AKBA on CD25 Expression

**Figure 4:**
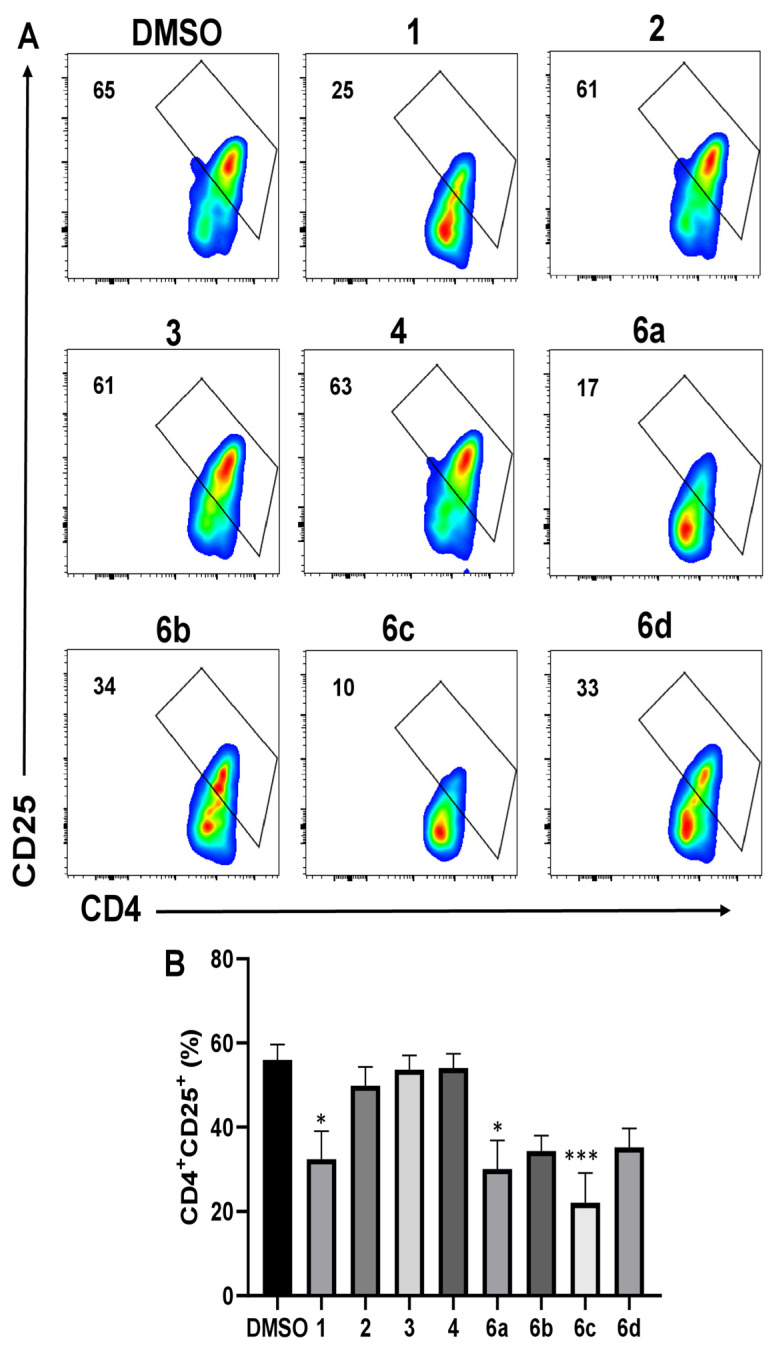
The effect of *β*-AKBA and derivative compounds at a concentration of 25 µM on the expression of CD25 in CD4^+^ T cells. Activated T cells were stained with mAbs and evaluated by flow cytometry. Representative flow cytometric plots showing the gating and percentage of CD25^+^ in CD4^+^ T cells in the presence of *β*-AKBA and derivative compounds (**A**). The overall percentages of CD4^+^CD25^+^ T-cell bar plots for all experiments performed (n = 8) are shown in (**B**). Compounds **1**, **6a**, and **6c** significantly reduced the CD25 T-cell activation marker on CD4^+^ T cells, compared with DMSO. * *p* < 0.05; *** *p* < 0.001.

**Figure 5:**
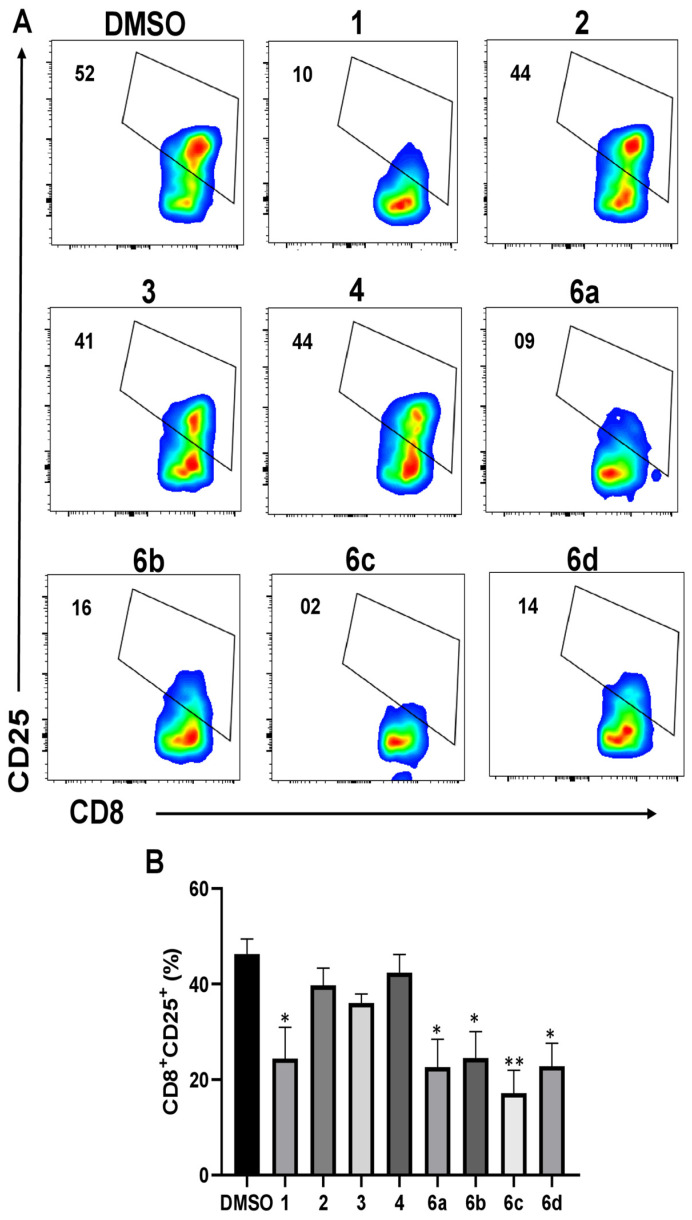
The effect of *β*-AKBA and derivative compounds at a concentration of 25 µM on the expression of CD25^+^ in CD8^+^ T cells. Activated T cells were stained with mAbs and evaluated by flow cytometry. Representative flow cytometric plots showing the gating and percentage of CD25^+^ in CD8^+^ T cells in the presence of *β*-AKBA and derivative compounds (**A**). The overall percentages of CD8^+^CD25^+^ T-cell bar plots for all experiments performed (n = 8) are shown in (**B**). Compounds **1**, **6a**, **6b**, **6c**, and **6d** significantly reduced the CD25 T-cell activation marker on CD8^+^ T cells, compared with DMSO. * *p* < 0.05; ** *p* < 0.01.

CD25 (IL-2 receptor alpha chain, IL2Rα) is a common surface marker of T-cell activation. Our previous work revealed that β-AKBA could regulate T-cell activation by reducing CD25 expression in CD4^+^ and CD8^+^ T cells [32]. To determine the effect of 1*H*-1,2,3-triazole derivatives of β-AKBA on T-cell activation, cells were stained with anti-human CD4, CD8, and CD25 antibodies. CD25^+^ levels were determined in CD4^+^ and CD8^+^ T-cell subpopulations, as shown in Supplementary Figure S1D,F,G. At the lower concentration of 12.5 µM, compound **1** (β-AKBA) and its 1H-1,2,3-triazole derivatives had no significant changes in CD25 expression in either CD4^+^ (Supplementary Figure S4) or CD8^+^ (Supplementary Figure S5) T cells. However, at the higher concentration of 25 µM, some compounds reduced CD25 CD4^+^ T cells (Figure 4A). More specifically, β-AKBA and compounds **6a**–**6d** reduced CD25^+^ levels in CD4^+^ T cells; however, this reduction was significant in the presence of β-AKBA, **6a**, and **6c** (Figure 4B). In CD8^+^ T cells, β-AKBA and compounds **6a**, **6b**, **6c**, and **6d** significantly reduced CD25^+^ levels (Figure 5A,B). Compared with β-AKBA, compound **6c** did not reduce the levels of CD25 expression in either CD4^+^ or CD8^+^ T cells (*p* values = 0.30 and 0.38, respectively). Overall, these compounds were able to inhibit T-cell activation, resulting in reduced proliferation ability. These findings highlight the promising therapeutic potential of 1H-1,2,3-triazole derivatives of β-AKBA in T-cell-mediated diseases.

## 3. Discussion

In recent years, numerous studies have revealed the significant biological activities of organic heterocyclic compounds with triazoles against inflammation and microbes. Notably, 1*H*-1,2,3-triazole and its derivatives demonstrate considerable pharmacological activities against conditions such as cancer, microbial infections, viral diseases, and inflammation [22,33]. To our knowledge, no studies to date have assessed the impact of 1*H*-1,2,3-triazole derivatives of *β*-AKBA on human T-cell modulation. In this study, we synthesized 1*H*-1,2,3-triazole derivatives of *β*-AKBA and evaluated their efficacy as immunomodulatory agents in human T cells.

This study demonstrated the effect of 1*H*-1,2,3-triazole derivatives of *β*-AKBA on T-cell activation and proliferation. The excessive or uncontrolled proliferation of T cells may result in chronic inflammation and inflammation-related diseases such as inflammatory bowel disease and rheumatoid arthritis. Therefore, the suppression of T-cell activation and proliferation can dampen overactive immune responses, resulting in the mitigation of inflammation and prevention of tissue damage, which is caused mainly by severe inflammation or T-cell-mediated autoimmunity. Our results revealed that the modified compounds linked with 1,2,3-triazole ring (**6a**, **6b**, and **6d**) have more ability to inhibit T-cell proliferation than *β*-AKBA, which may be due to the presence of a 1,2,3-triazole ring. This ring may enhance the structural stability, demonstrating more effects in the biological systems, compared to *β*-AKBA alone. 1,2,3-triazoles can interact with biological targets such as receptors and enzymes involved in pathological signaling pathways via non-covalent interactions [34].

Many studies have shown that inhibiting T-cell proliferation and activation is a promising therapeutic approach for treating autoimmune disorders and inflammatory diseases [35,36,37]. BAs, including *β*-AKBA, play beneficial roles by inhibiting inflammatory cytokines, including IL-1*β*, IL-2, IL-6, IFN-γ, and tumor necrosis factor-alpha (TNF-α) in both in vitro and in vivo experiments [38,39]. Therefore, *β*-AKBA may play an important role in modulating Th1 and Th2 cell differentiation through shifting proinflammatory Th1 cytokines toward anti-inflammatory Th2 cytokines, which is a crucial step for reducing chronic inflammation and helping to improve symptoms.

1*H*-1,2,3-triazole derivatives can act as anti-inflammatory agents, as reported in previous studies [40]. For instance, 1,2,3-triazole-linked pyrrolobenzodiazepine could inhibit the activity of the cyclooxygenase (COX) enzyme, which plays a pivotal role in producing prostaglandin (PG), which is a crucial mediator in various pathophysiological processes including inflammation [41]. A recent study by Jahan H. et al. revealed that triazole derivatives exhibit remarkable inhibitory activity against key mediators of inflammation, such as cyclooxygenase-2 (COX-2) and its product, prostaglandin E2 (PGE2), which in turn play important roles in the inflammation cascade [42]. They reported that indole or carbazole linked to 1*H*-1,2,3-triazole derivatives can inhibit the activation of the advanced glycation end-product (AGE)–reactive oxygen species (ROS)–nuclear factor kappa B (NF-κB) signaling pathway in monocytes, resulting in the inhibition of proinflammatory COX-2/PGE2 [42]. Another study revealed that 1,2,3-triazole-based benzoxazolinones are potential anti-inflammatory and antinociceptive agents inhibiting COX-2 [43]. Moreover, Tae Woo Kim et al. reported that phenyl-linked 1*H*-1,2,3-triazole derivatives play a key role in inhibiting inflammation [39]. These studies support our findings by linking bioactive natural products or anti-inflammatory agents with a triazole ring enhancing their biological activity. Furthermore, our findings showed the immunoinhibitory effects of *β*-AKBA (**1**) and its derivative compounds on T-cell activation. The combination of *β*-AKBA with 1*H*-1,2,3-triazole could enhance the pharmacological effects of compounds **6a**, **6b**, and **6d** by regulating immune activation. These compounds show the greatest inhibition of T-cell activation by modulating the expression of the CD25 activation marker. The possibility that compounds may disrupt the signaling pathways linked with T-cell activation either via extracellular stimulatory signals or via inducing intracellular signaling cascades downstream of the TCR complex. This regulation may affect key immune modulators such as cell surface receptors and effector cytokines, leading to inhibiting T-cell activation.

Compound 6c, which contains a trifluoromethyl group (-CF3), also significantly reduced T-cell proliferation and activation, but with cytotoxic effect. Previous studies have shown that trifluoromethyl groups can regulate biological activity [44,45]. They can act as an anticancer and anti-inflammatory agent by inhibiting immune activity, likely through the regulation of inflammatory signaling pathways that modulate the activation of T cells. It is possible that *β*-AKBA-containing 1*H*-1,2,3-triazole and trifluoromethyl groups (compound **6c**) could impact T-cell viability. Therefore, a series of *β*-AKBA-linked 1-*H*-1,2,3-triazole derivatives may have inhibitory effects on key mediators of inflammation that are involved in inflammatory diseases or autoimmune diseases.

## 4. Materials and Methods

### 4.1. Preparation of Boswellic Acid (BA) Cluster

**Scheme 1:**
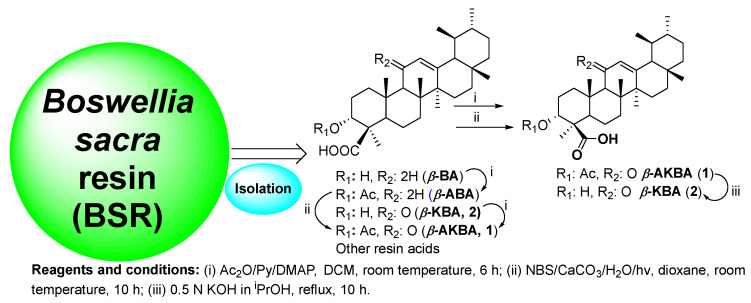
Gram-scale production of *β*-AKBA (**1**) isolated from BSR.

*B. sacra* was collected from different locations in Dhofar province, Oman (August 2016), and was formally identified by a reliable partner (Mr. Saleh Al-Amri). The sample (No: B4SHR-01/2016) was deposited with the Herbarium of Natural and Medical Sciences Research Center (NMSRC), University of Nizwa, Oman. The extraction of *β*-AKBA has been described in our previous study [46]. *β*-AKBA (compound **1**) was obtained as white crystals from the oleogum resin of the *B. sacra* plant using a modification of Jauch’s protocol (Scheme 1) [47].

### 4.2. Synthesis of 1H-1,2,3-Triazole Derivatives of β-AKBA (**6a**–**6d**)

**Scheme 2:**
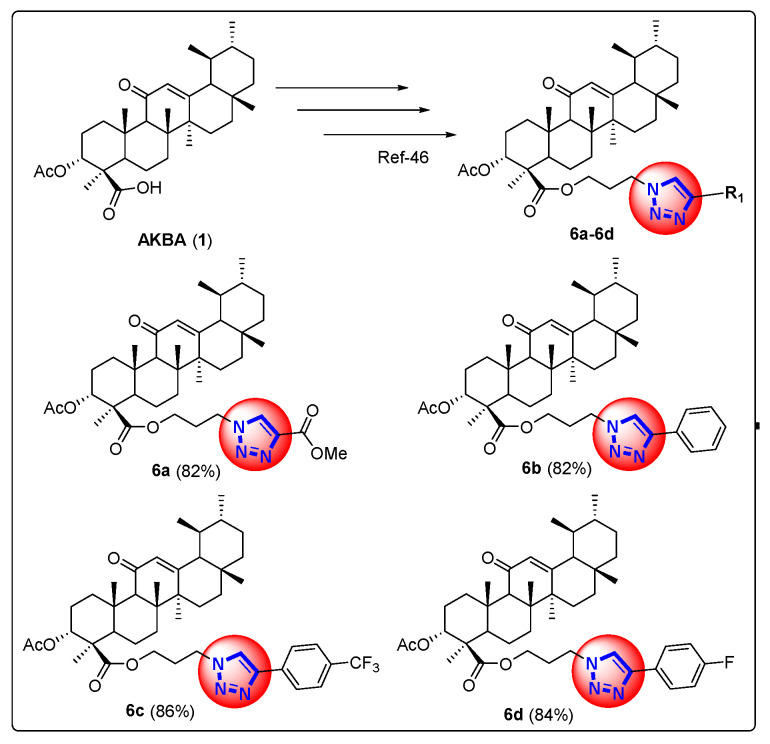
Synthesis of 1*H*-1,2,3-triazole derivatives of *β*-AKBA (**6a**–**6d**).

The experiments were conducted in dry reaction vessels under a dry nitrogen (N) atmosphere. All chemicals and reagents were purchased from Sigma-Aldrich (St. Louis, MO, USA). The solvents were purified and dried according to standard procedures. The synthesis of 1*H*-1,2,3-triazole derivatives of *β*-AKBA (**2**–**4** and **6a**–**6d**) was performed at NMSRC, University of Nizwa, Oman. The synthetic scheme of 1*H*-1,2,3-triazole derivatives of *β*-AKBA (**6a**–**6d**) from *β*-AKBA (**1**) is depicted in Scheme 2. All NMR data of these compounds have been described in our previously published article [46]. The synthesis of all derivative compounds was prepared according to our previously published methods [46].

### 4.3. Antibodies and Reagents

RPMI-1640 medium with L-glutamine and the antibiotic/penicillin-streptomycin (Pen-Strep) were purchased from Gibco-Life Technologies (Grand Island, NY, USA; Cat. No. 15140122). Sodium azide (NaN_3_, 99%) was obtained from Acros Organics ((Morris Plains, NJ, USA)) and fetal bovine serum (FBS) from GibcoTM (Grand Island, NY, USA; Cat. No. 26140079), while dimethyl sulfoxide (DMSO) and Histopaque^®^-1077 were obtained from Sigma-Aldrich (St. Louis, MO, USA; Cat. No. D4540 and 10771, respectively). Fixable viability dye eFluor-450 (FVD-450, Thermo-Fisher Scientific, eBioscienceTM, San Diego, CA, USA; Cat. No. 65-0863-18) and carboxy-fluorescein diacetate succinimidyl-ester (CFSE, Thermo Fisher Scientific, eBioscience (San Diego, CA, USA); Cat. No. 65-0850-84) were used to measure cell viability and proliferation, respectively. Soluble anti-human CD3 antibody (clone: OKT3; eBioscience; 16-0037-85) and anti-human CD28 antibody (clone: CD28.2; eBioscience; Cat. No. 16-0289-85) were used for cell stimulation. Monoclonal antibodies (mAbs), including anti-CD4-PE-Cyanine7 (Clone: RPA-T4; eBioscience; Cat. No. 25-0049-42), anti-CD8a-Super Bright-702 (clone: OKT-8; eBioscience; Cat. No. 67-0086-42), and anti-CD25-APC-eFluor-780 (clone: CD25-4E3; eBioscience; Cat. No. 47-0257-42) were used for cell surface staining.

### 4.4. Isolation of Peripheral Blood Mononuclear Cells

Blood samples were collected from healthy donors following informed consent (study No.: BA-2023–01). PBMCs were isolated from blood using Histopaque^®^-1077 (Sigma-Aldrich, St. Louis, MO, USA), according to the manufacturer’s instructions. Briefly, blood was layered onto Histopaque solution and centrifuged at 400× *g* without braking for 30 min. The mononuclear cell interphase was collected and washed using RPMI-medium. PBMCs were re-suspended in a freezing medium, aliquoted into cryovials, and stored in liquid nitrogen.

### 4.5. T-Cell Proliferation Assays

PBMCs were labeled with CFSE according to the manufacturer’s instructions. After thawing, the PBMCs were washed twice with RPMI-medium, and the pellet was re-suspended at a cell number of 1 × 10^6^ cells/mL in PBS. The cells were stained with CFSE (2 µM) and incubated in the dark for 10 min at room temperature. Then, the staining process was halted by adding an ice-cold culture medium (CM, RPMI, 10% FBS, and 1% pen/strep). The stained cells were washed and re-suspended in CM and then stimulated with 0.25 μg/mL soluble anti-CD3 and 0.25 μg/mL soluble anti-CD28 antibodies in a 96-U-bottom tissue culture plate at a concentration of 1 × 10^5^ cells/well. The cells were treated with either different concentrations (12.5 and 25 µM) of *β*-AKBA, compounds **2**, **3**, **4**, **6a**, **6b**, **6c**, and **6d**; or equivalent amounts of negative control (DMSO). The treated cells were placed in an incubator at 37 °C with carbon dioxide (5%) and 95% humidity. After three days of incubation, T-cell proliferation was measured by CFSE dilution using Northern Lights flow cytometry (Cytek Biosciences, Inc., Fremont, CA, USA).

### 4.6. Cell Staining and Flow Cytometry

After culture, the cells were collected, washed with cold PBS, and resuspended in 0.1 mL of staining buffer (PBS with 0.1% NaN_3_ and 2% FBS). Then, the cells were stained with FVD-450 at 4 °C for 30 min to measure viability and gate live cells in flow cytometric analyses. Additionally, antibodies against CD4 and CD8 were added to measure T-cell subpopulations, while an anti-CD25 antibody was used to measure T-cell activation. Following staining, the cells were washed with cold PBS, re-suspended in a staining buffer (300 µL), and immediately run on a flow cytometry. The data were analyzed using FlowJo version-10 software (TreeStar, Ashland, OR, USA).

### 4.7. Statistical Analyses

The results were analyzed using GraphPad Prism v8 (GraphPad software, San Diego, CA, USA) and presented as the mean ± standard error of the mean (SEM) for each group. The normality of the datasets was assessed using the Shapiro–Wilk test. One-way ANOVA and Kruskal–Wallis analysis were used to compare the differences between groups. A *p* value of less than 0.05 was considered statistically significant. Data are represented as follows: * *p* < 0.05, ** *p* < 0.01, *** *p* < 0.001, and **** *p* < 0.0001.

## 5. Conclusions

We reported the design, synthesis, and effects of 1*H*-1,2,3-triazole derivatives of *β*-AKBA from *B. sacra* resin on T-cell proliferation and activation. Interestingly, some of the synthesized derivatives exhibited highly potent antiproliferative activity on T cells at a concentration of 25 µM. The inhibition of T-cell proliferation and reduction in the expression of CD25 activation marker by *β*-AKBA and **6a**, **6b**, and **6d** could have anti-inflammatory potentials. The attaching of a 1*H*-1,2,3-triazole to *β*-AKBA appears to increase its activity, likely due to interaction with key biological targets that are required in the inflammatory signaling pathway. These findings can contribute to understanding how the modification of bioactive natural compounds with 1*H*-1,2,3-triazole can enhance their biological activities. Therefore, this study may contribute to the growing field of research on pharmacological activities.

## Data Availability

The datasets used and/or analyzed during the current study are available from the corresponding author on reasonable request.

## Supplementary Materials

The following supporting information can be downloaded at: https://www.mdpi.com/article/10.3390/ph17121650/s1, Figure S1. Gating strategy of flow cytometry. Representative flow cytometric plots show gating of lymphocytes (**A**), singlet cells (**B**), live cells (**C**), CFSE+ nonproliferating/ CFSE- proliferating singlet lymphocytes (**D**), CD4^+^ and CD8^+^ T cells within live cells (**E**), CD25^+^ percentage within CD4^+^ T cells (**F**), and CD25^+^ percentage within CD8^+^ T cells (**G**). Figure S2. The effect of β-AKBA and 1H-1,2,3-triazole derivatives at 12.5 μM concentration on T cell viability after three days of stimulation was determined by FVD-450 dye and flow cytometry. Representative flow cytometric plots are shown in (**A**), and overall viability bar plots for all experiments performed (n = 6) are shown in (**B**). Figure S3. The effect of β-AKBA and 1H-1,2,3-triazole derivatives at 12.5 μM concentration on T cell proliferation was determined by CFSE loss. Representative flow cytometric plots are shown in (**A**), and overall cell proliferation bar plots for all experiments performed (n = 8) are shown in (**B**). Figure S4. Effect of β-AKBA and their derivatives at 12.5 μM concentration on expression of CD25 in CD4^+^ T cells. Activated T cells were stained with monoclonal antibodies and analyzed by flow cytometry. Representative flow cytometric plots show gating and percentage of CD4^+^CD25^+^ T cells in the presence of β-AKBA and derivative compounds (**A**). The overall percentages of CD4^+^CD25^+^ T cells bar plots for all experiments performed (n = 8) are shown in (**B**). Figure S5. Effect of β-AKBA and their derivatives at 12.5 μM concentration on expression of CD25^+^ in CD8^+^ T cells. Activated T cells were stained with monoclonal antibodies and analyzed by flow cytometry. Representative flow cytometric plots show gating and percentage of CD8^+^ CD25^+^ T cells in the presence of β-AKBA and derivative compounds (**A**). The overall percentages of CD8^+^CD25^+^ T cells bar plots for all experiments performed (n = 8) are shown in (**B**). Figure S6. Gram-scale synthesis of boswellic acids (BAs, β-AKBA, 1) from Boswellia sacra resin (BSR). Reagents and conditions: (i) Ac2O/Py/DMAP, CH2Cl2, RT, 6 h; (ii) NBS/CaCO3/H2O/hν, dioxane, RT, 10 h; (iii) 0.5 N KOH in iPrOH, reflux, 10 h.
