# Supplementary material for: Effects of 1H-1,2,3-Triazole Derivatives of 3-O-Acetyl-11-Keto-Beta-Boswellic Acid from Boswellia sacra Resin on T-Cell Proliferation and Activation
Source: Pharmaceuticals (Basel). 2024 Dec 8;17(12):1650. doi: 10.3390/ph17121650 (PMC11728835; doi:10.3390/ph17121650)
Supplement: Supplementary file 1 [file pharmaceuticals-17-01650-s001.zip › NMR supplementary data.pdf]

Table of contents **Page**

**S1**: ^{1}H-NMR spectrum (600 MHz, $CDCl_3$) of compound **2**	2
**S2**: ^{13}C-NMR spectrum (150 MHz, $CDCl_3$) of compound **2**	2
**S3**: HRMS spectrum of compound **2**	3
**S4**: ^{1}H-NMR spectrum (600 MHz, $CDCl_3$) of compound **3**	3
**S5**: ^{13}C-NMR spectrum (150 MHz, $CDCl_3$) of compound **3**	4
**S6**: HRMS spectrum of compound **3**	4
**S7**: ^{1}H-NMR spectrum (600 MHz, $CDCl_3$) of compound **4**	5
**S8**: ^{13}C-NMR spectrum (150 MHz, $CDCl_3$) of compound **4**	5
**S9**: HRMS spectrum of compound **4**	6
**S10**: ^{1}H-NMR spectrum (600 MHz, $CDCl_3$) of compound **6a**	6
**S11**: ^{13}C-NMR spectrum (150 MHz, $CDCl_3$) of compound **6a**	7
**S12**: HRMS spectrum of compound **6a**	7
**S13**: ^{1}H-NMR spectrum (600 MHz, $CDCl_3$) of compound **6b**	8
**S14**: ^{13}C-NMR spectrum (150 MHz, $CDCl_3$) of compound **6b**	8
**S15**: HRMS spectrum of compound **6b**	9
**S16**: ^{1}H-NMR spectrum (600 MHz, $CDCl_3$) of compound **6c**	9
**S17**: ^{13}C-NMR spectrum (150 MHz, $CDCl_3$) of compound **6c**	10
**S18**: ^{19}F-NMR spectrum (564 MHz, $CDCl_3$) of compound **6c**	10
**S19**: HRMS spectrum of compound **6c**	11
**S20**: ^{1}H-NMR spectrum (600 MHz, $CDCl_3$) of compound **6d**	11
**S21**: ^{13}C-NMR spectrum (150 MHz, $CDCl_3$) of compound **6d**	12
**S22**: ^{19}F-NMR spectrum (564 MHz, $CDCl_3$) of compound **6d**	12
**S23**: HRMS spectrum of compound **6d**	13

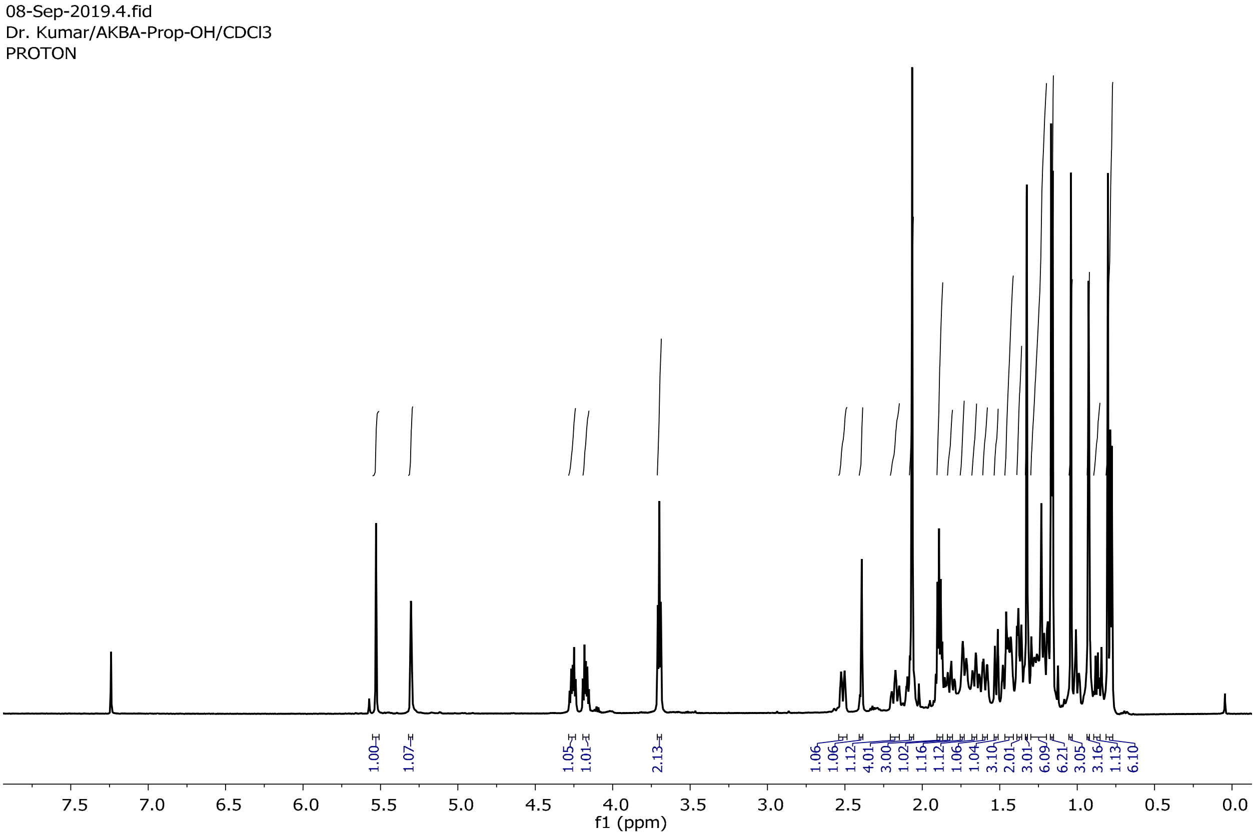

S1: ^{1}H-NMR spectrum (600 MHz, $CDCl_3$) of compound **2**

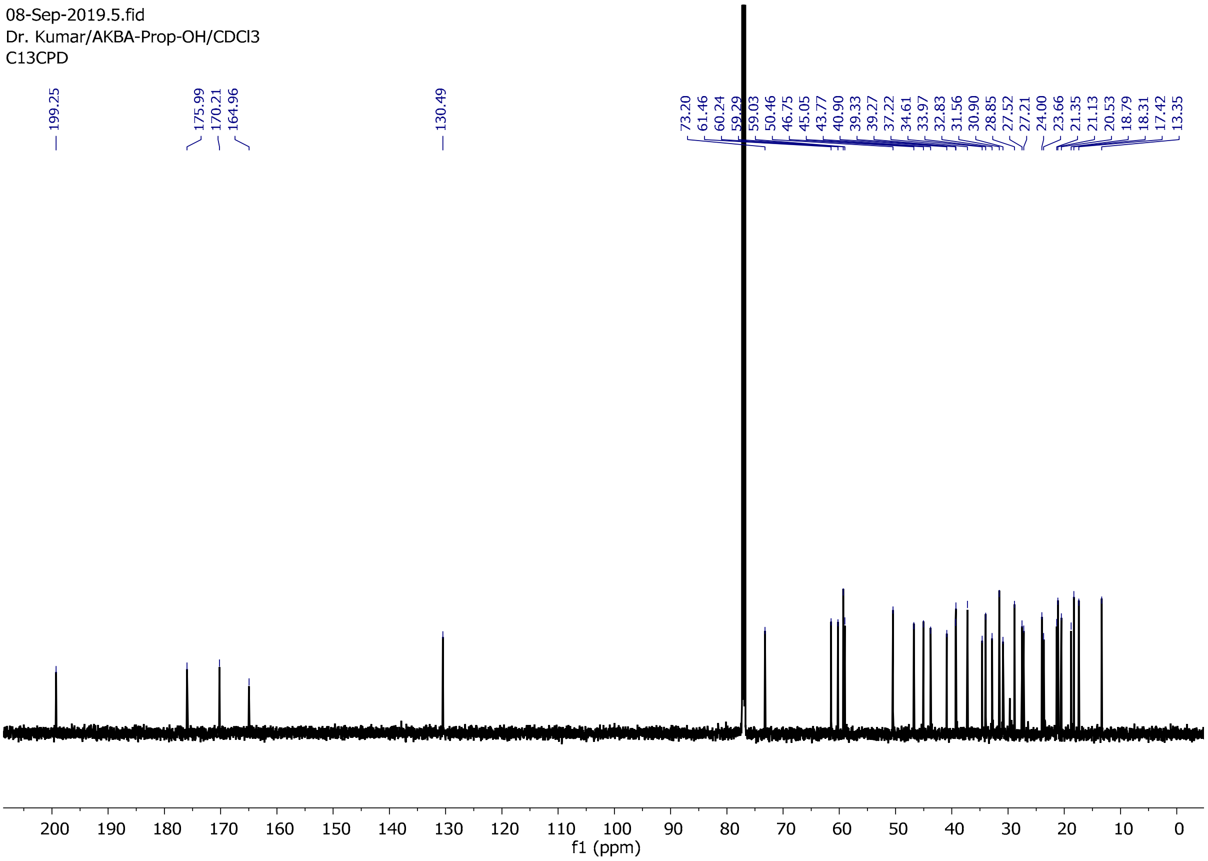

S2: ^{13}C-NMR spectrum (150 MHz, $CDCl_3$) of compound **2**

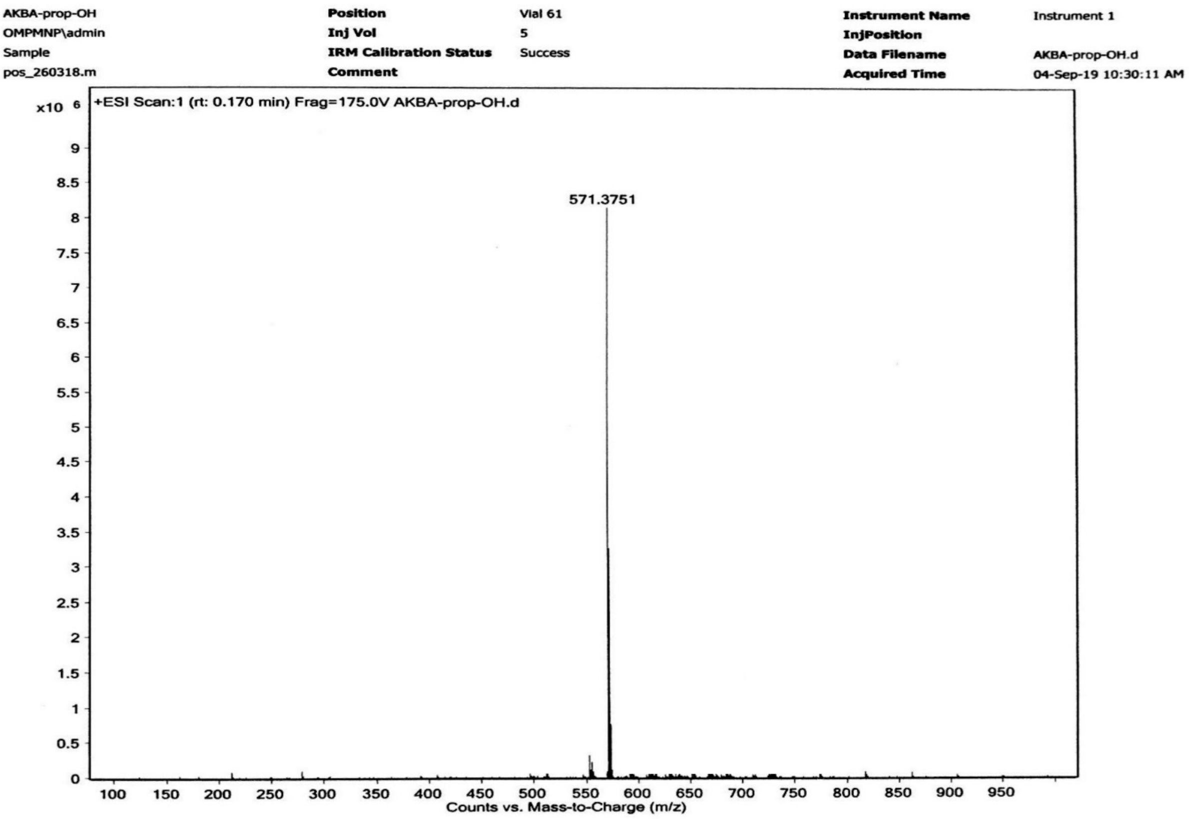

S3: HRMS spectrum of compound **2**

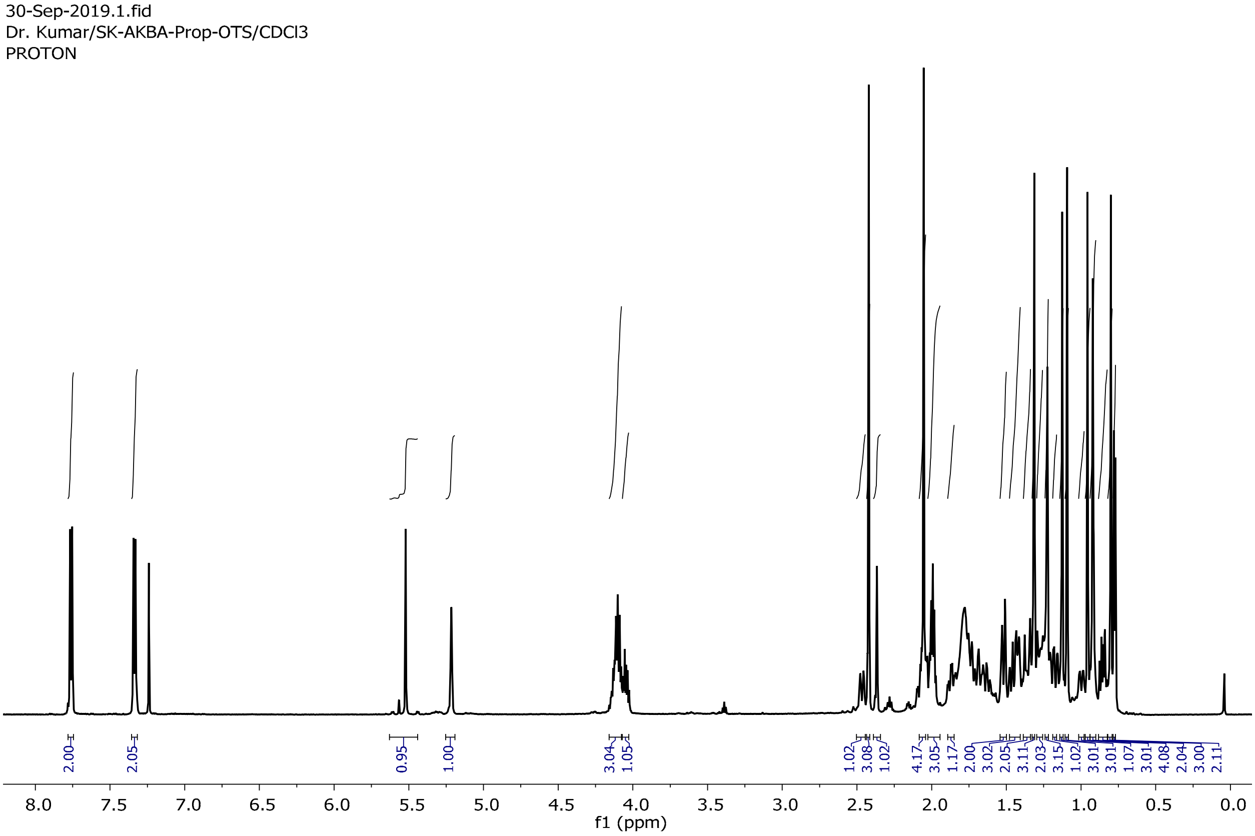

S4: ^{1}H-NMR spectrum (600 MHz, $CDCl_3$) of compound **3**

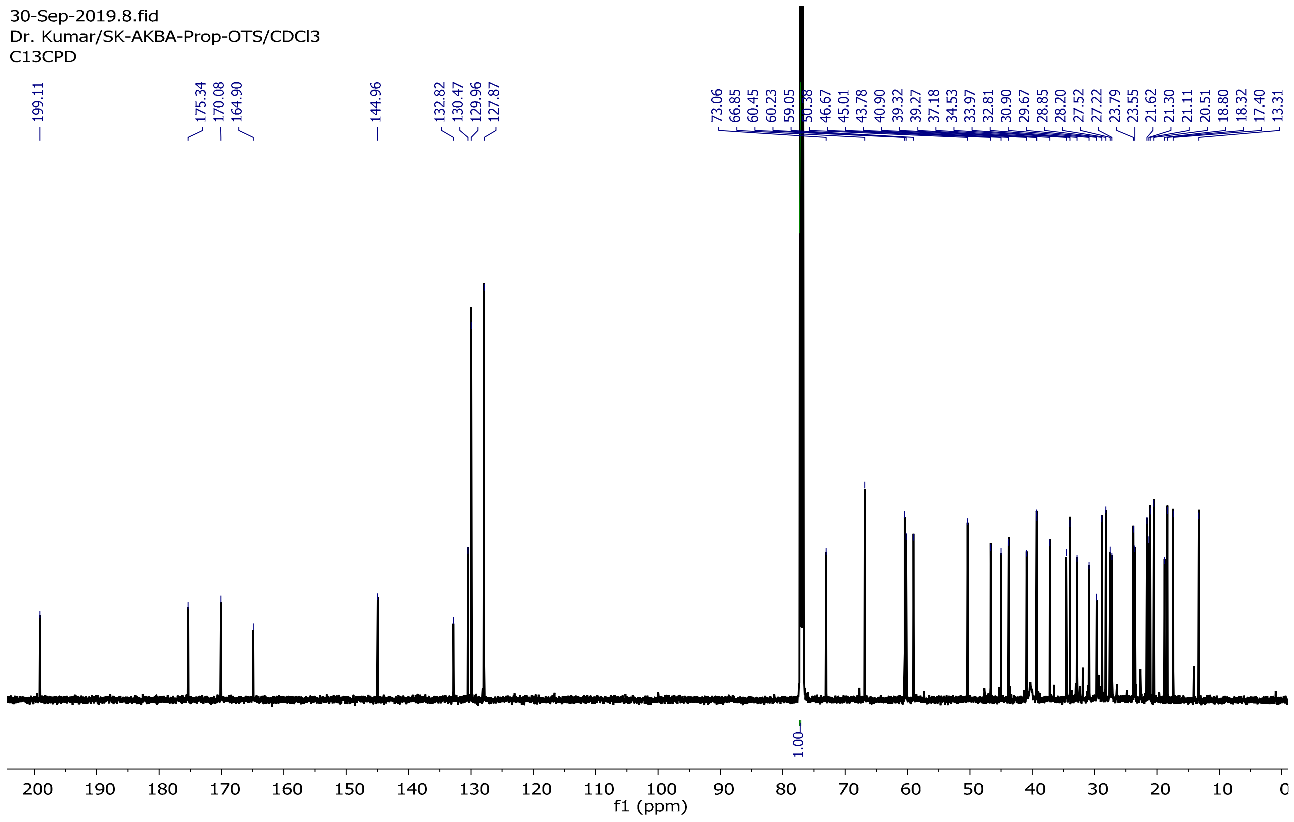

S5: ^{13}C-NMR spectrum (150 MHz, $CDCl_3$) of compound **3**

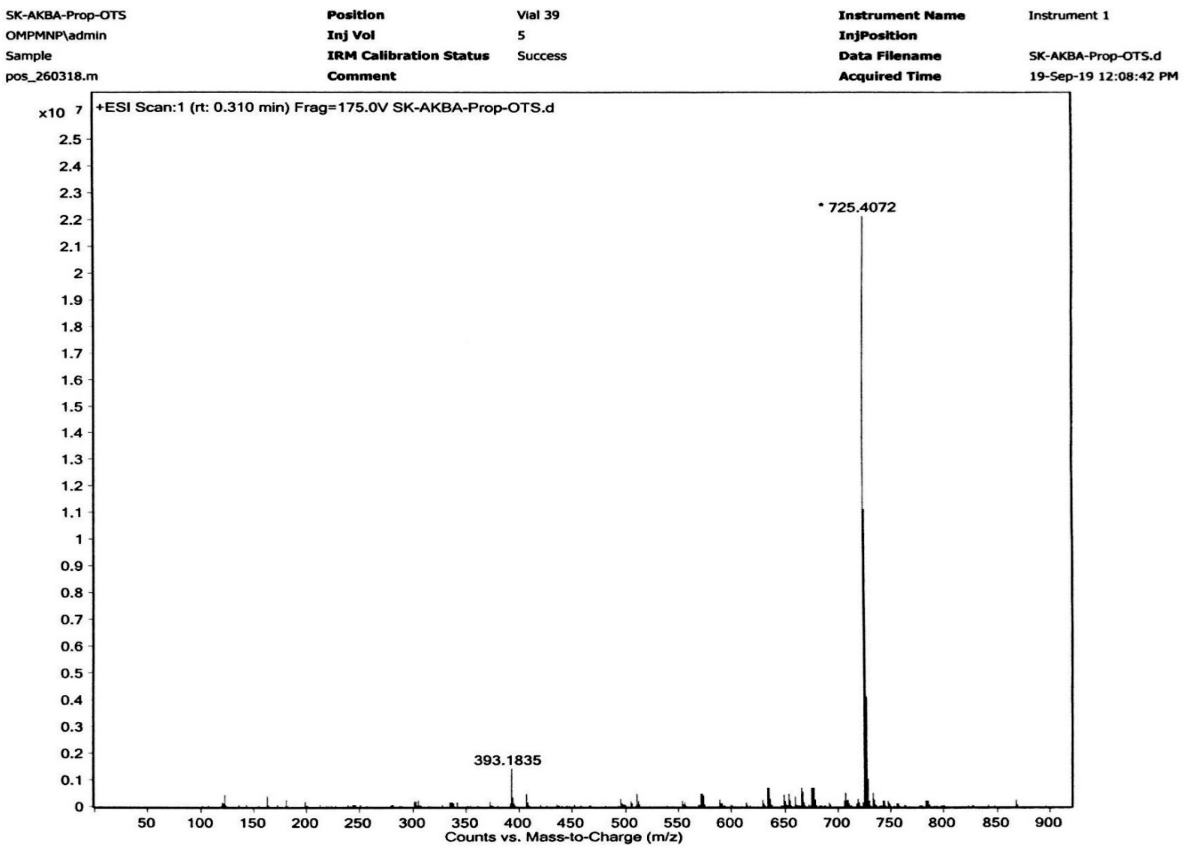

S6: HRMS spectrum of compound **3**

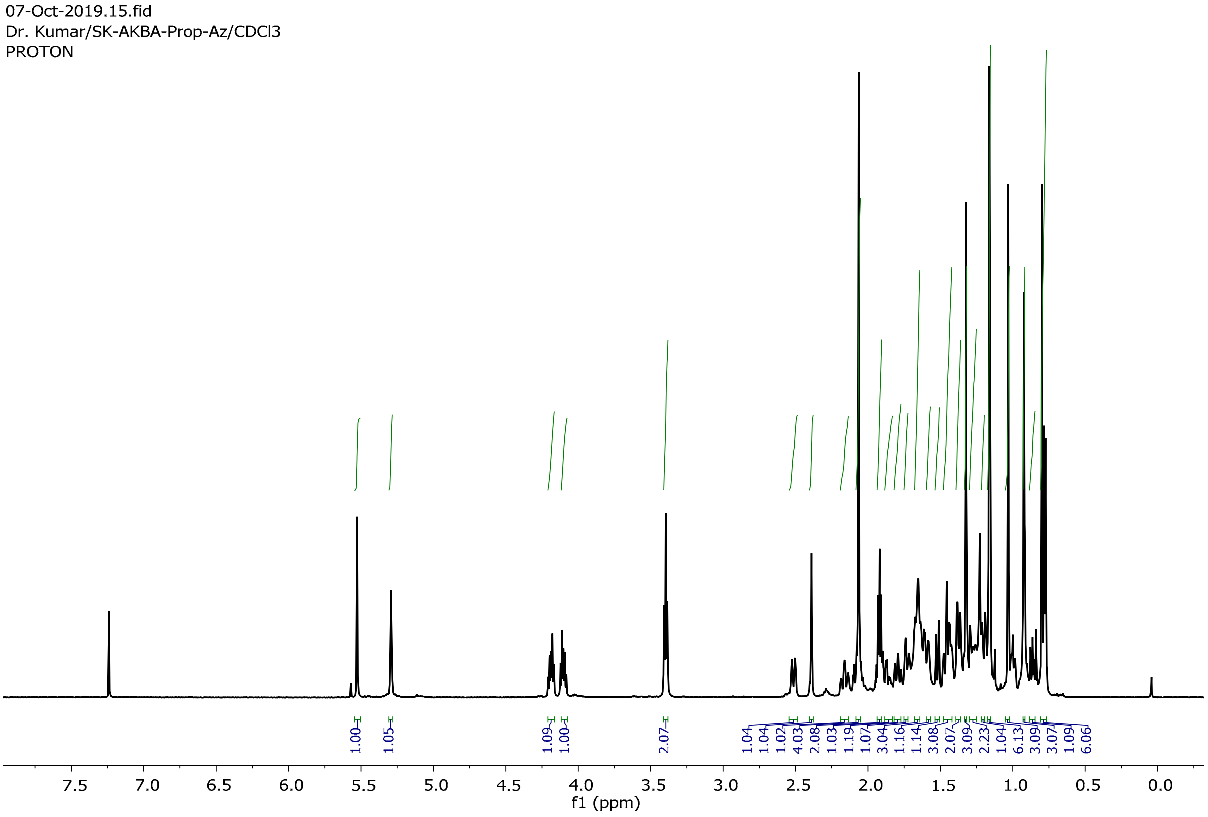

S7: ^{1}H-NMR spectrum (600 MHz, $CDCl_3$) of compound **4**

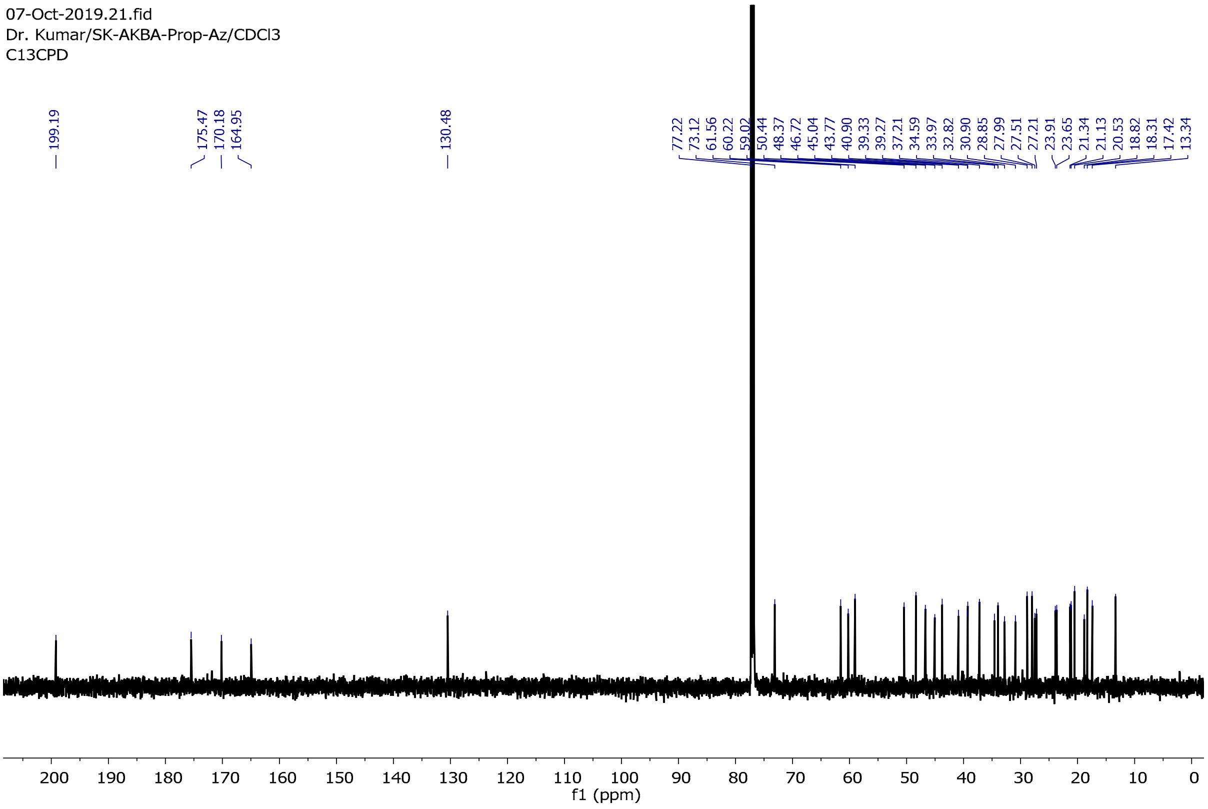

S8: ^{13}C-NMR spectrum (150 MHz, $CDCl_3$) of compound **4**

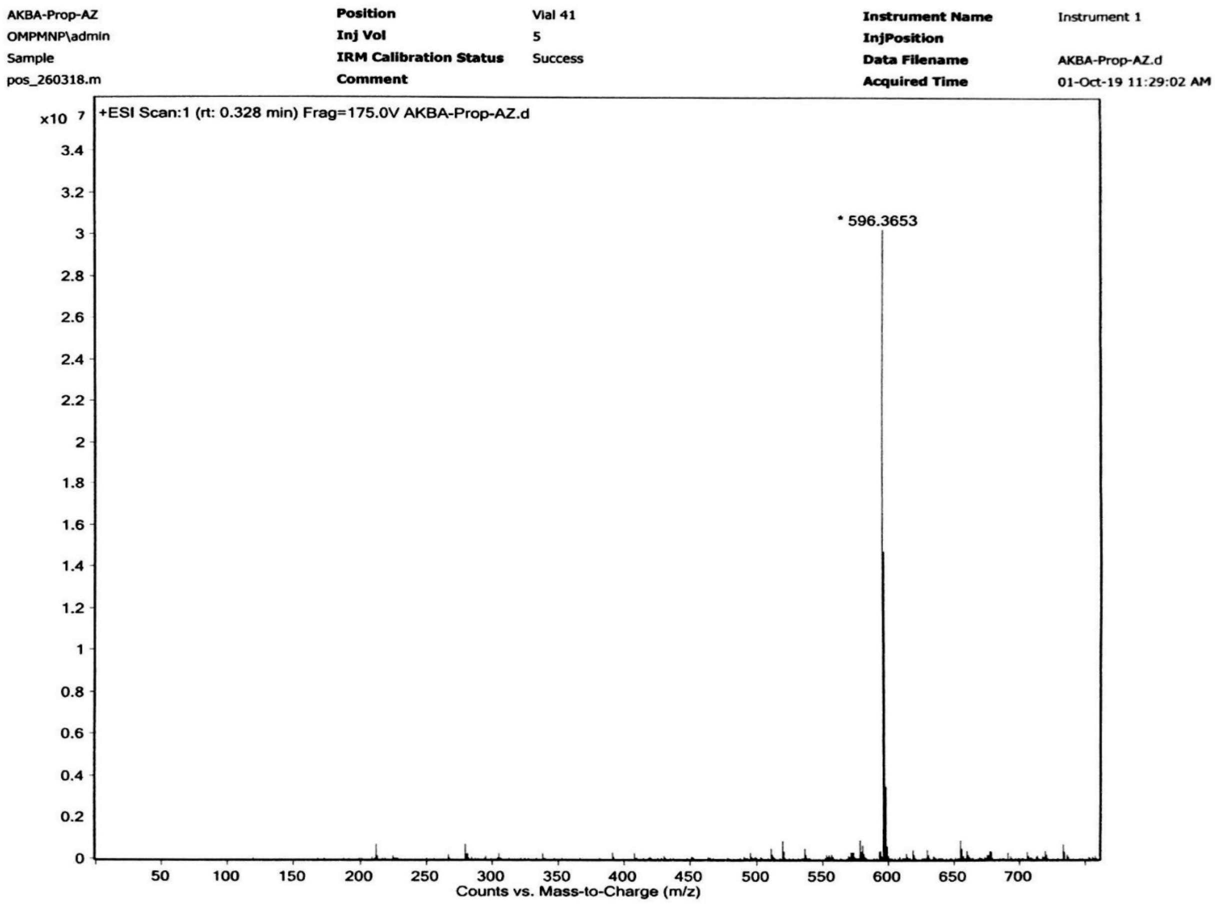

S9: HRMS spectrum of compound **4**

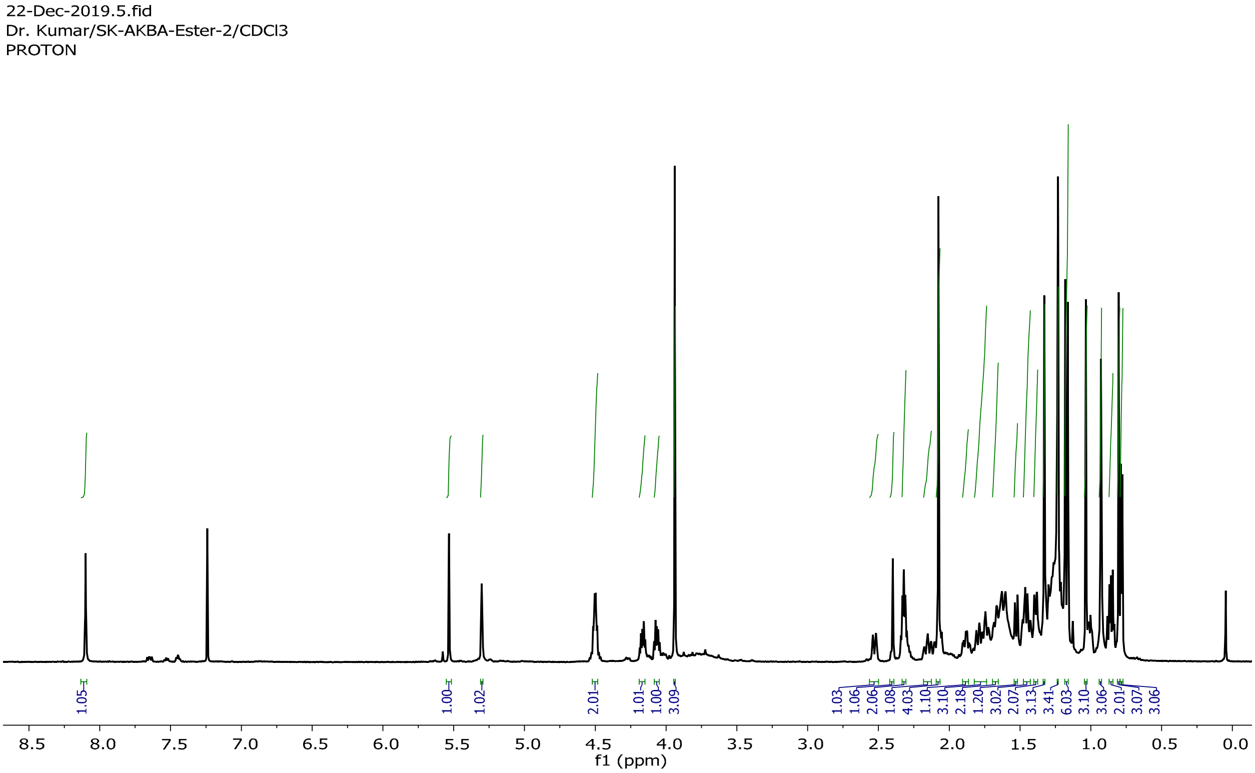

S10: ^{1}H-NMR spectrum (600 MHz, $CDCl_3$) of compound **6a**

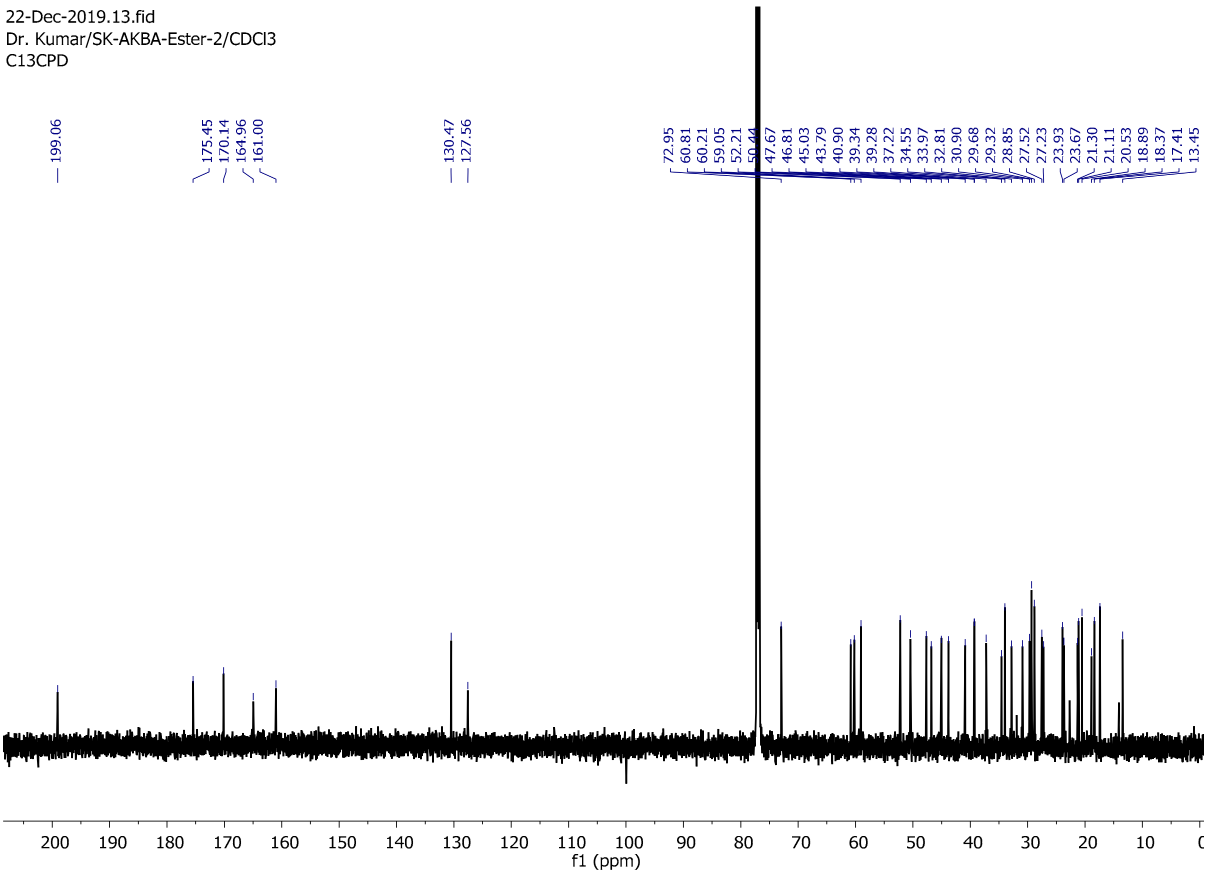

S11: ^{13}C-NMR spectrum (150 MHz, $CDCl_3$) of compound **6a**

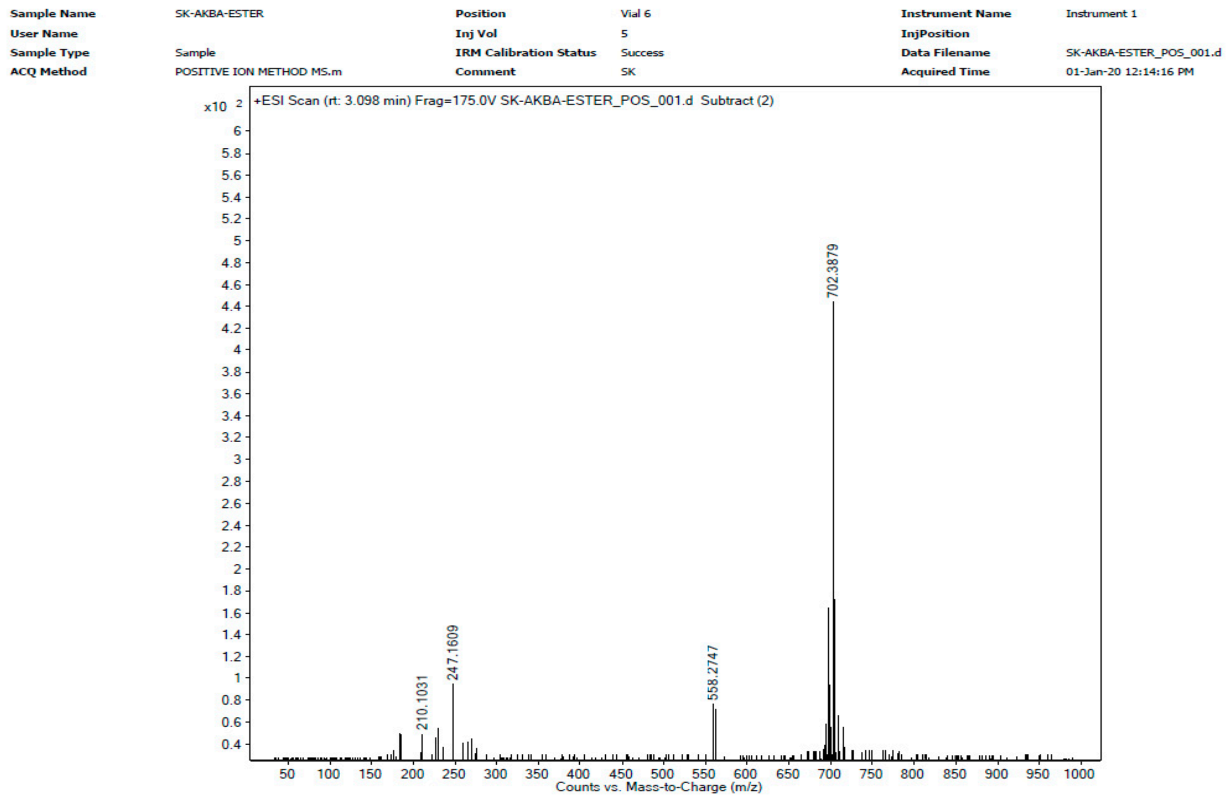

S12: HRMS spectrum of compound **6a**

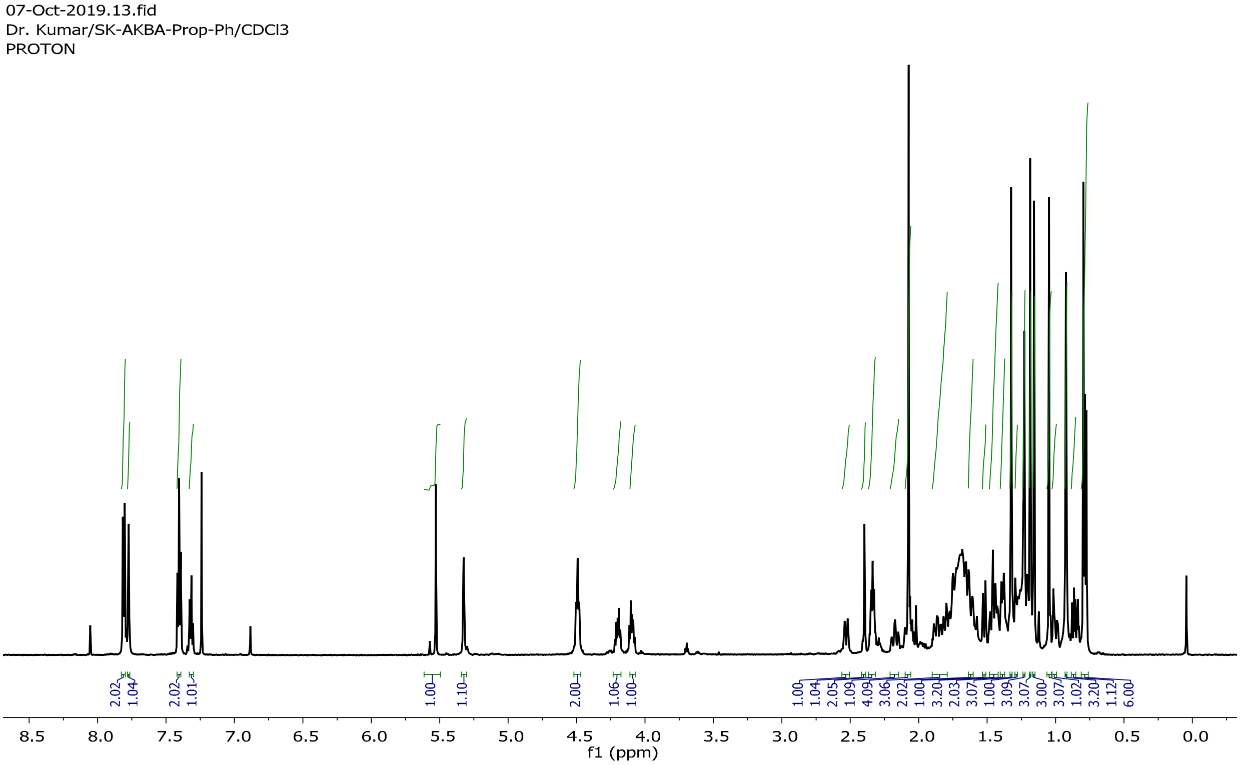

S13: ^{1}H-NMR spectrum (600 MHz, $CDCl_3$) of compound **6b**

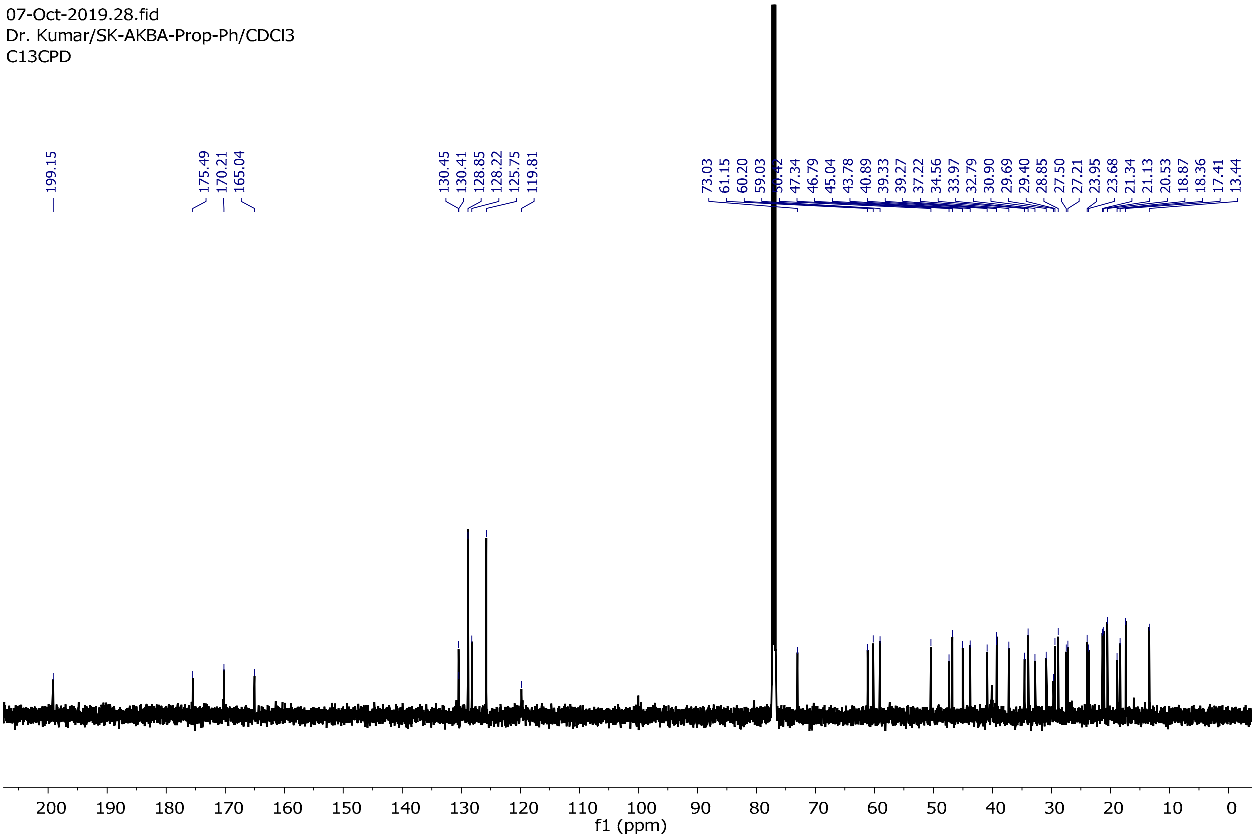

S14: ^{13}C-NMR spectrum (150 MHz, $CDCl_3$) of compound **6b**

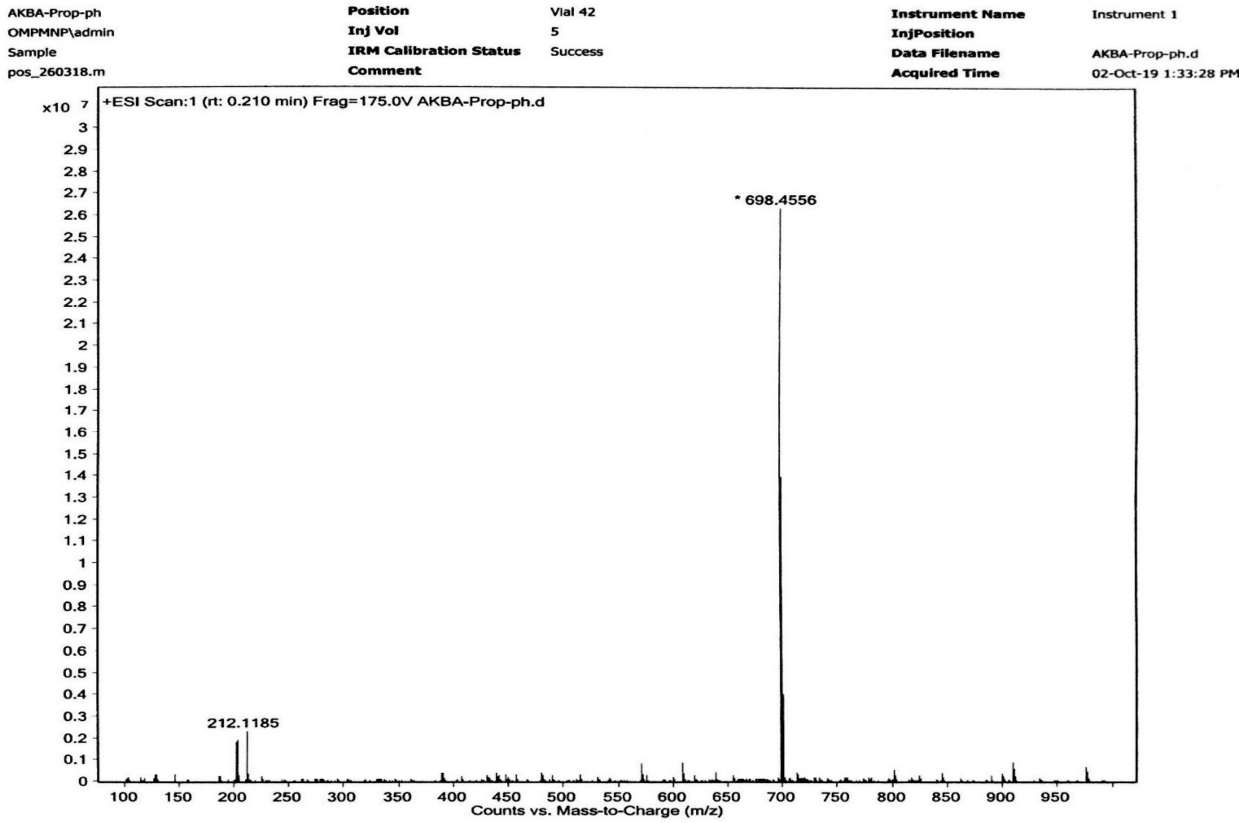

S15: HRMS spectrum of compound **6b**

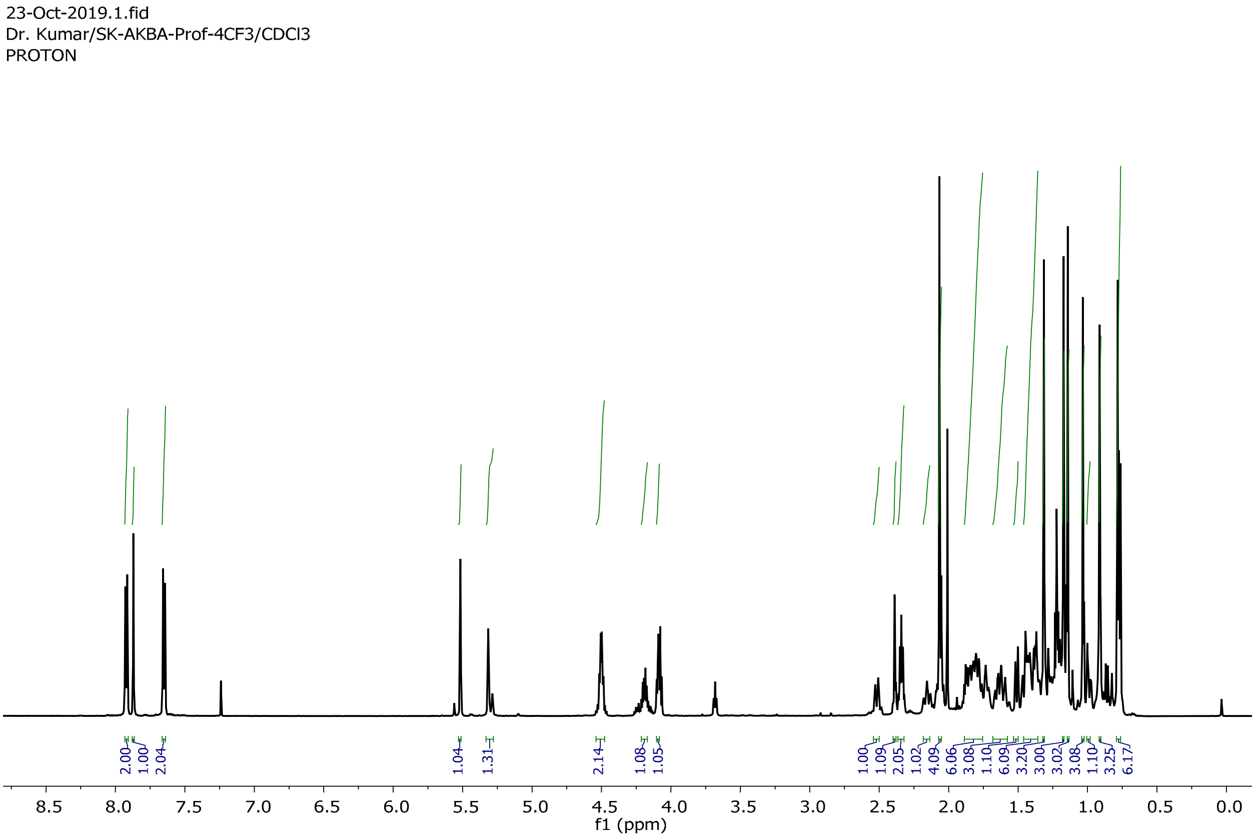

S16: ^{1}H-NMR spectrum (600 MHz, $CDCl_3$) of compound **6c**

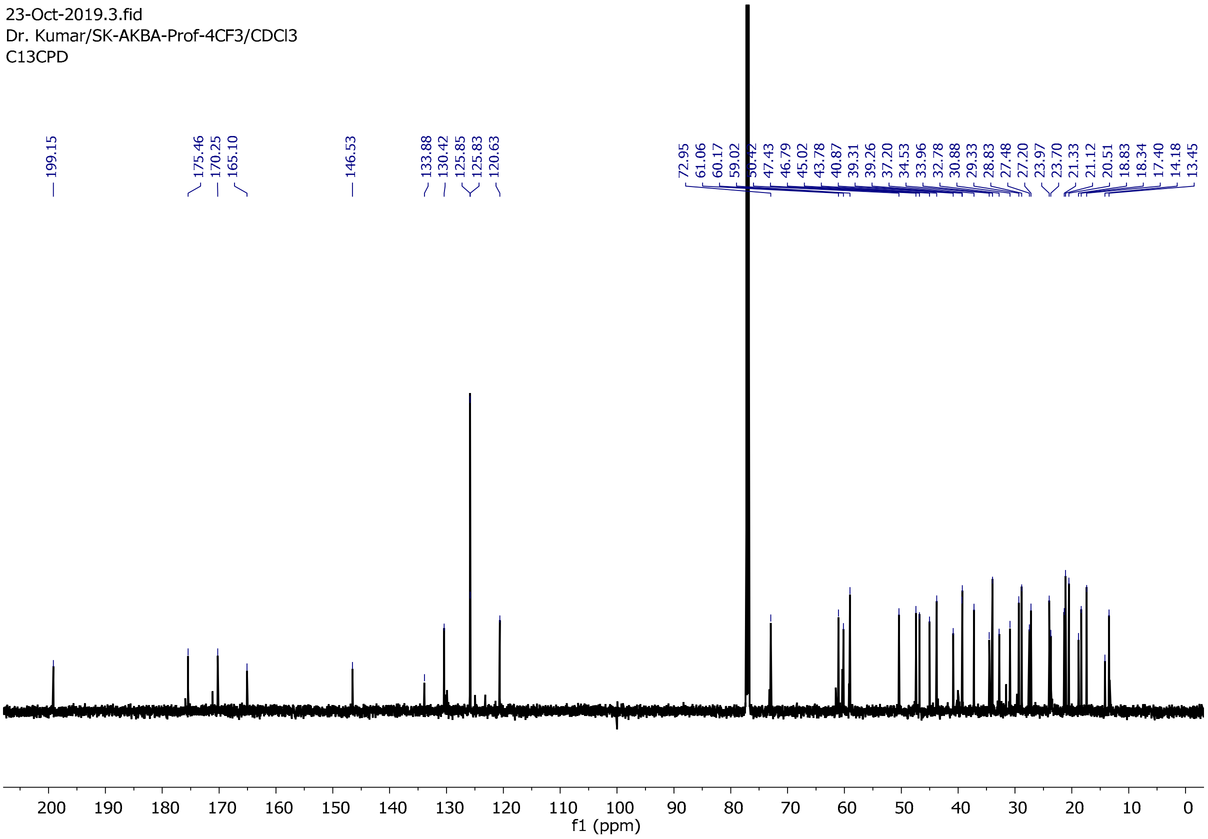

S17: ^{13}C-NMR spectrum (150 MHz, $CDCl_3$) of compound **6c**

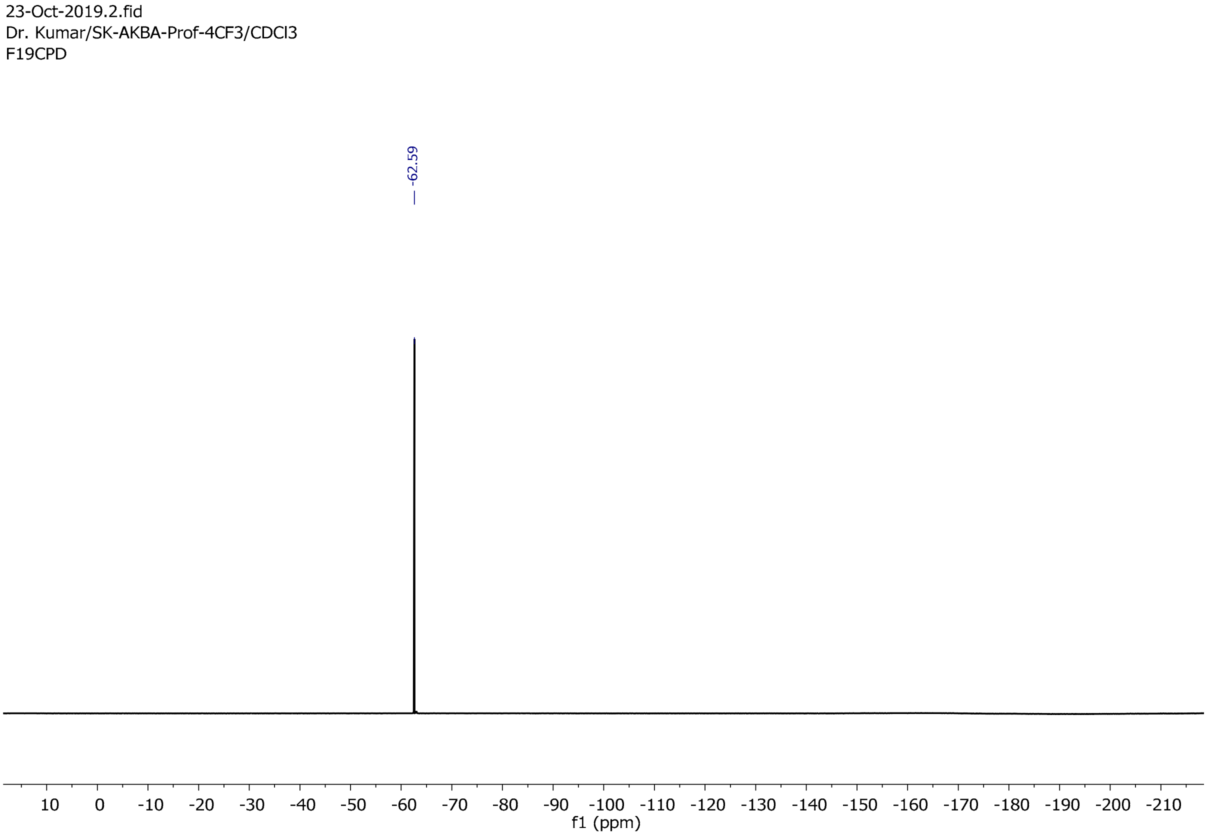

S18: ^{19}F-NMR spectrum (564 MHz, $CDCl_3$) of compound **6c**

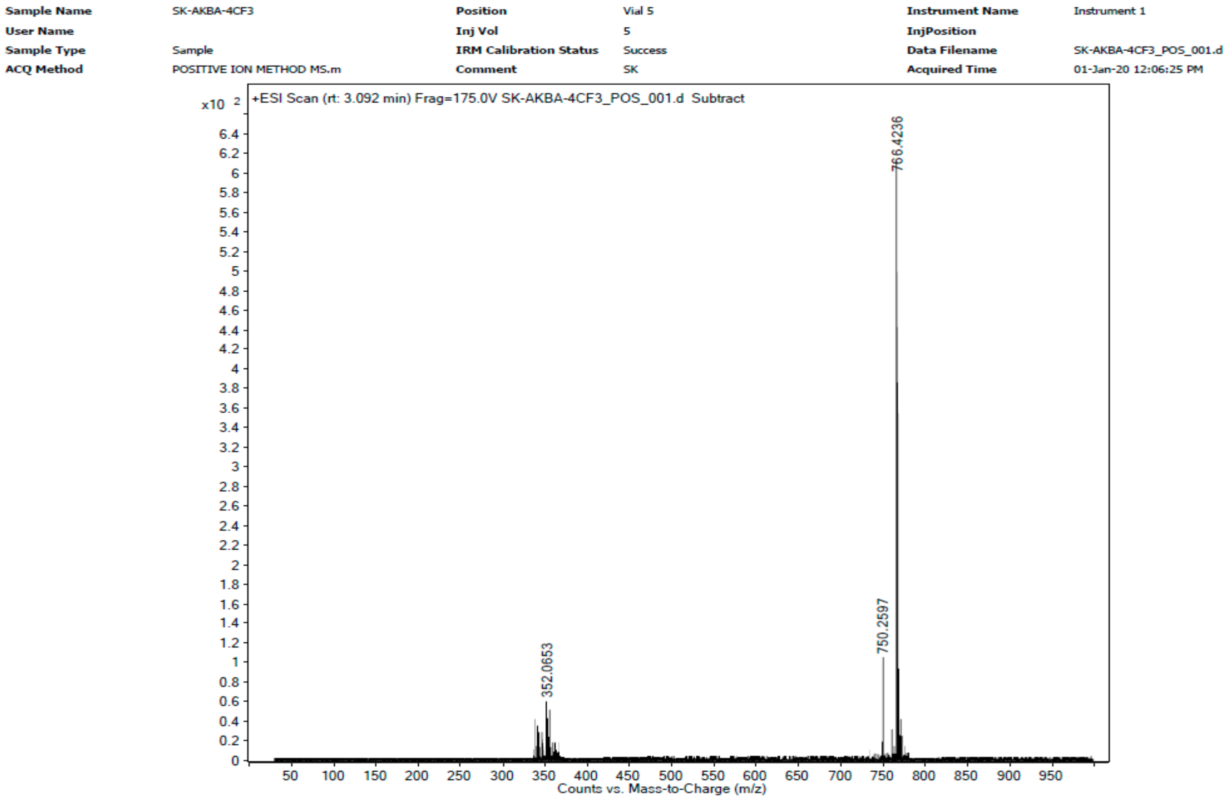

S19: HRMS spectrum of compound **6c**

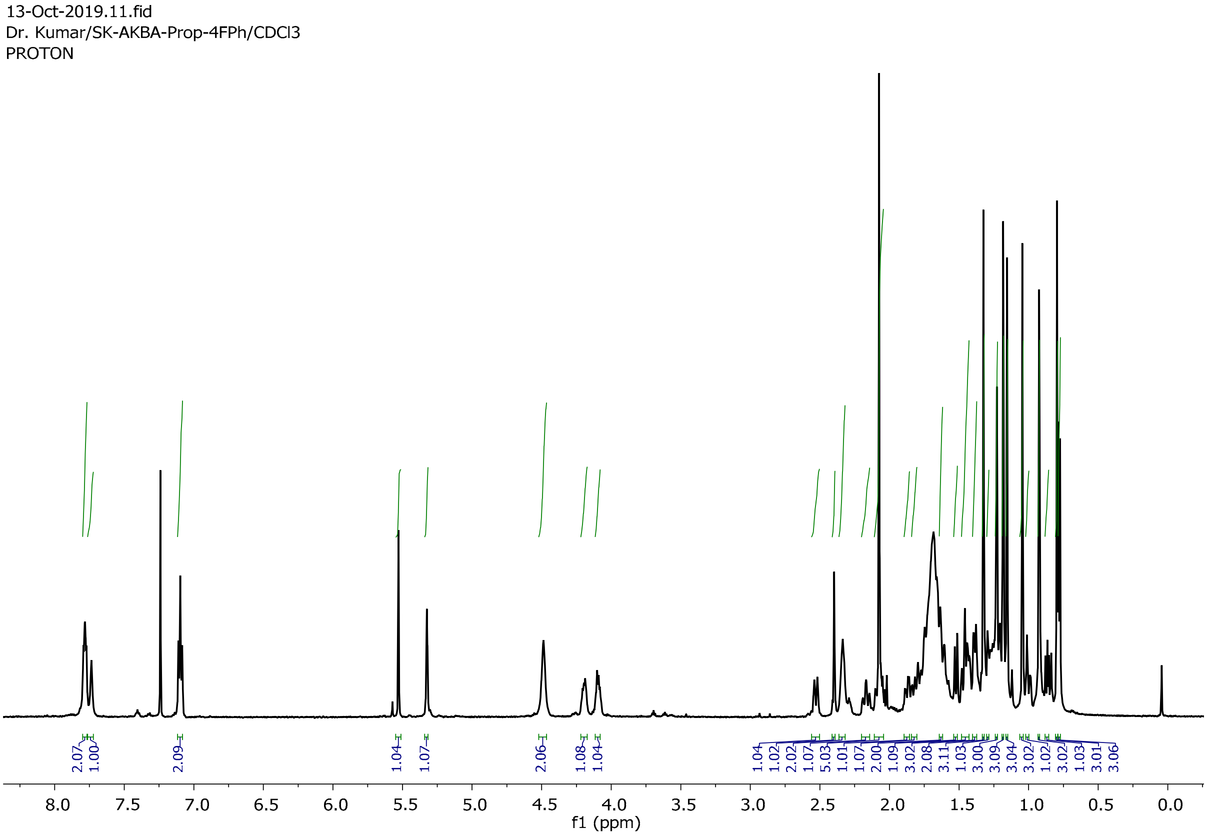

S20: ^{1}H-NMR spectrum (600 MHz, $CDCl_3$) of compound **6d**

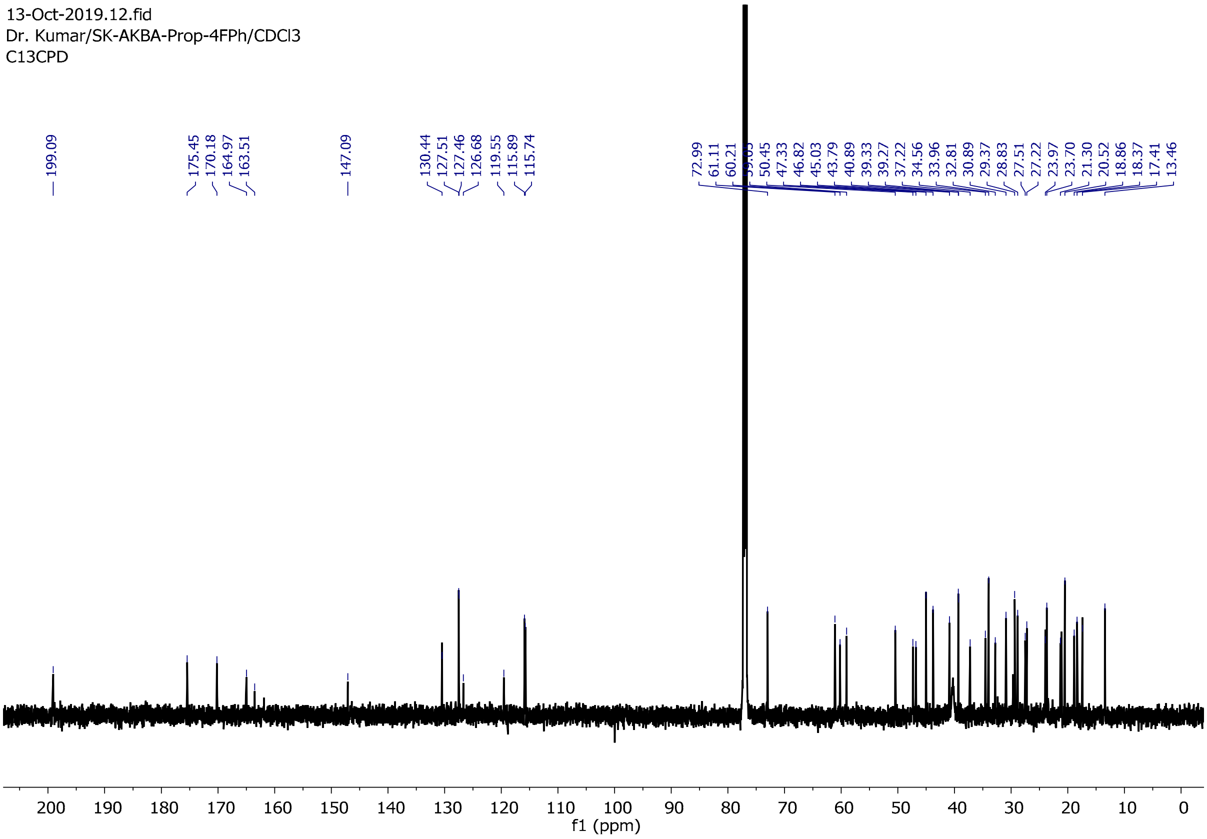

S21: ^{13}C-NMR spectrum (150 MHz, $CDCl_3$) of compound **6d**

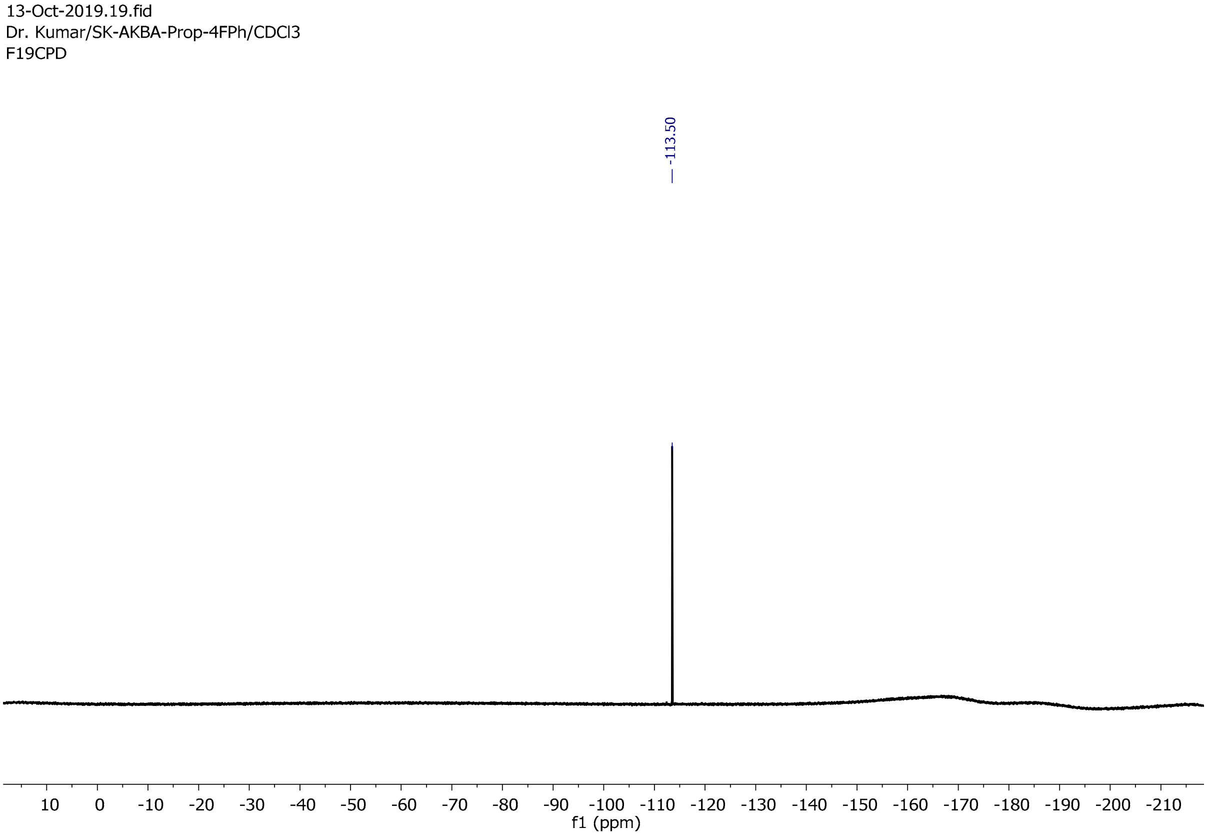

S22: ^{19}F-NMR spectrum (564 MHz, $CDCl_3$) of compound **6d**

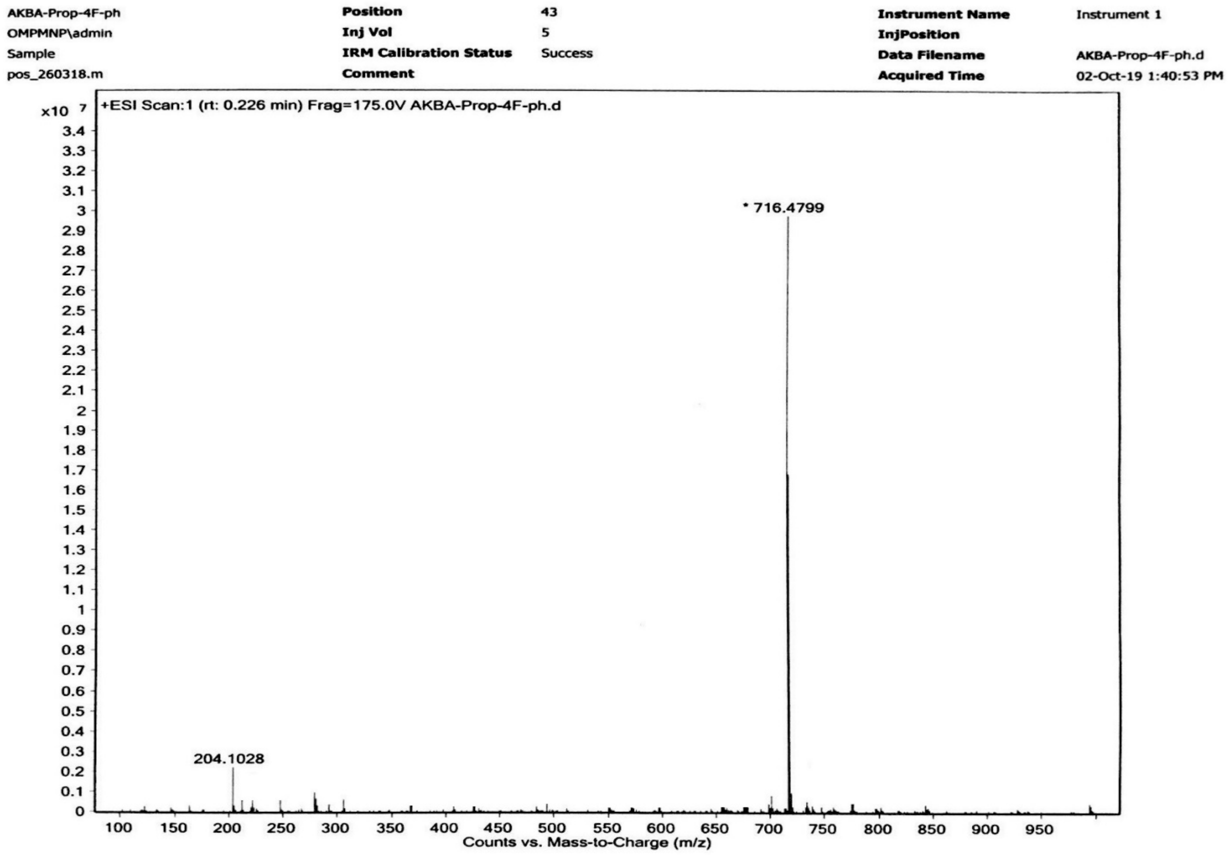

S23: HRMS spectrum of compound **6d**
