# Supplementary material for: Effects of 1H-1,2,3-Triazole Derivatives of 3-O-Acetyl-11-Keto-Beta-Boswellic Acid from Boswellia sacra Resin on T-Cell Proliferation and Activation
Source: Pharmaceuticals (Basel). 2024 Dec 8;17(12):1650. doi: 10.3390/ph17121650 (PMC11728835; doi:10.3390/ph17121650)
Supplement: Supplementary file 1 [file pharmaceuticals-17-01650-s001.zip › Supplementary Figures.pdf]

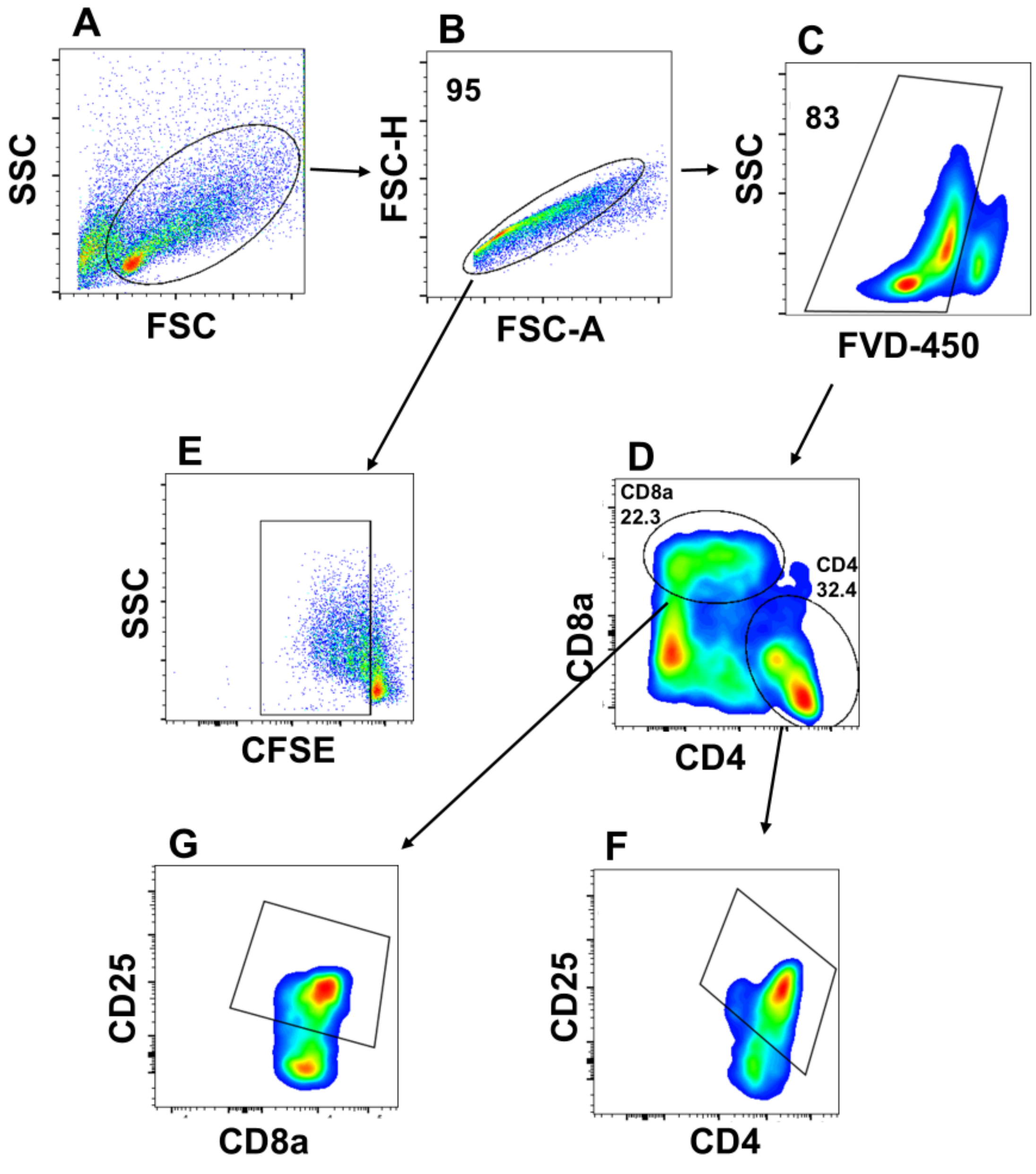

Supplementary Figure S1. Gating strategy of flow cytometry. Representative flow cytometric plots show gating of lymphocytes (**A**), singlet cells (**B**), live cells (**C**), CFSE^{+} nonproliferating/ CFSE^{-} proliferating singlet lymphocytes (**D**), CD4^{+} and CD8^{+} T cells within live cells (**E**), CD25^{+} percentage within CD4^{+} T cells (**F**), and CD25^{+} percentage within CD8^{+} T cells (**G**).

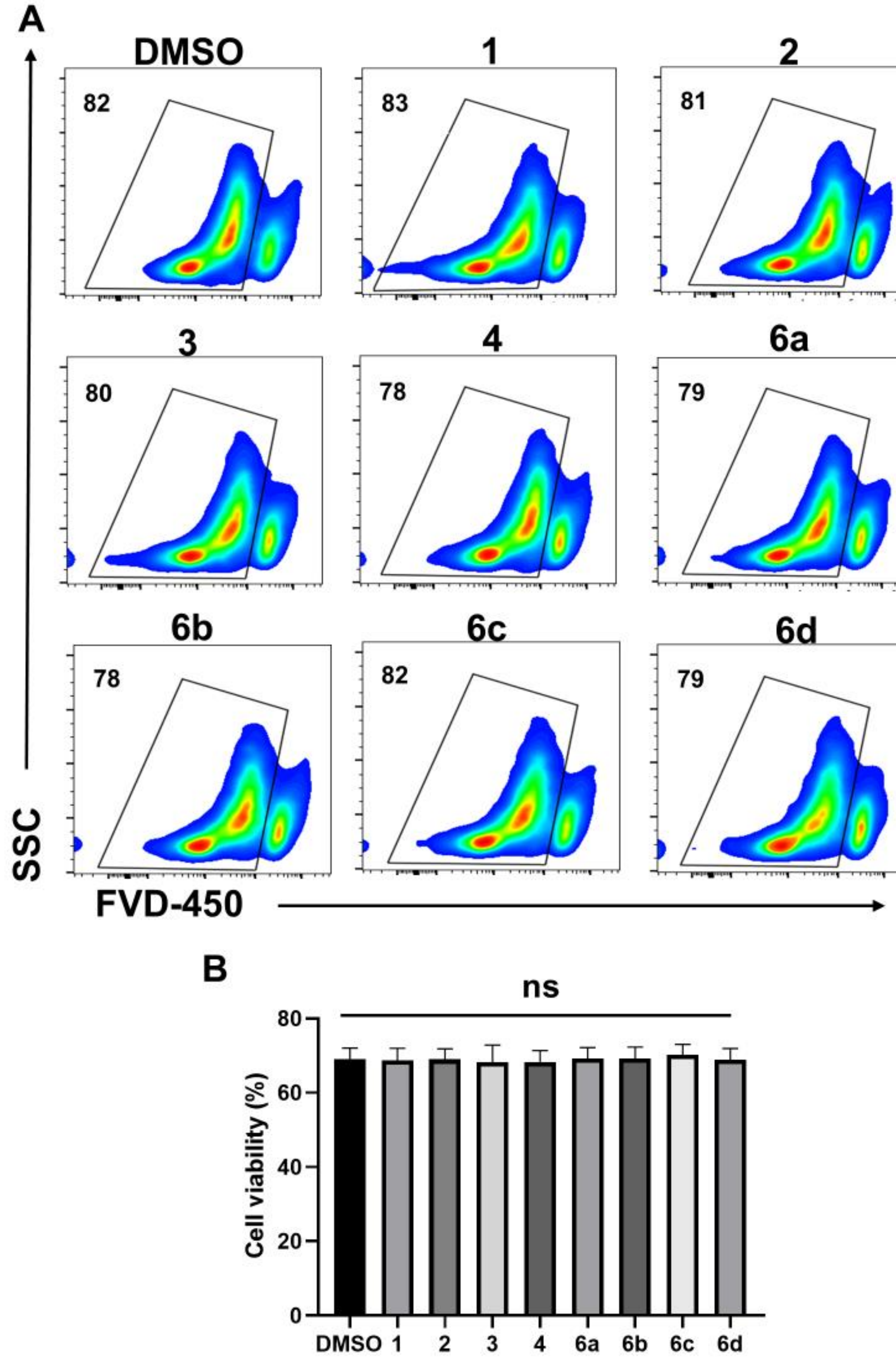

Supplementary Figure S2. The effect of *β*-AKBA and 1*H*-1,2,3-triazole derivatives at 12.5 μM concentration on T cell viability after three days of stimulation was determined by FVD-450 dye and flow cytometry. Representative flow cytometric plots are shown in (A), and overall viability bar plots for all experiments performed (n = 6) are shown in (B).

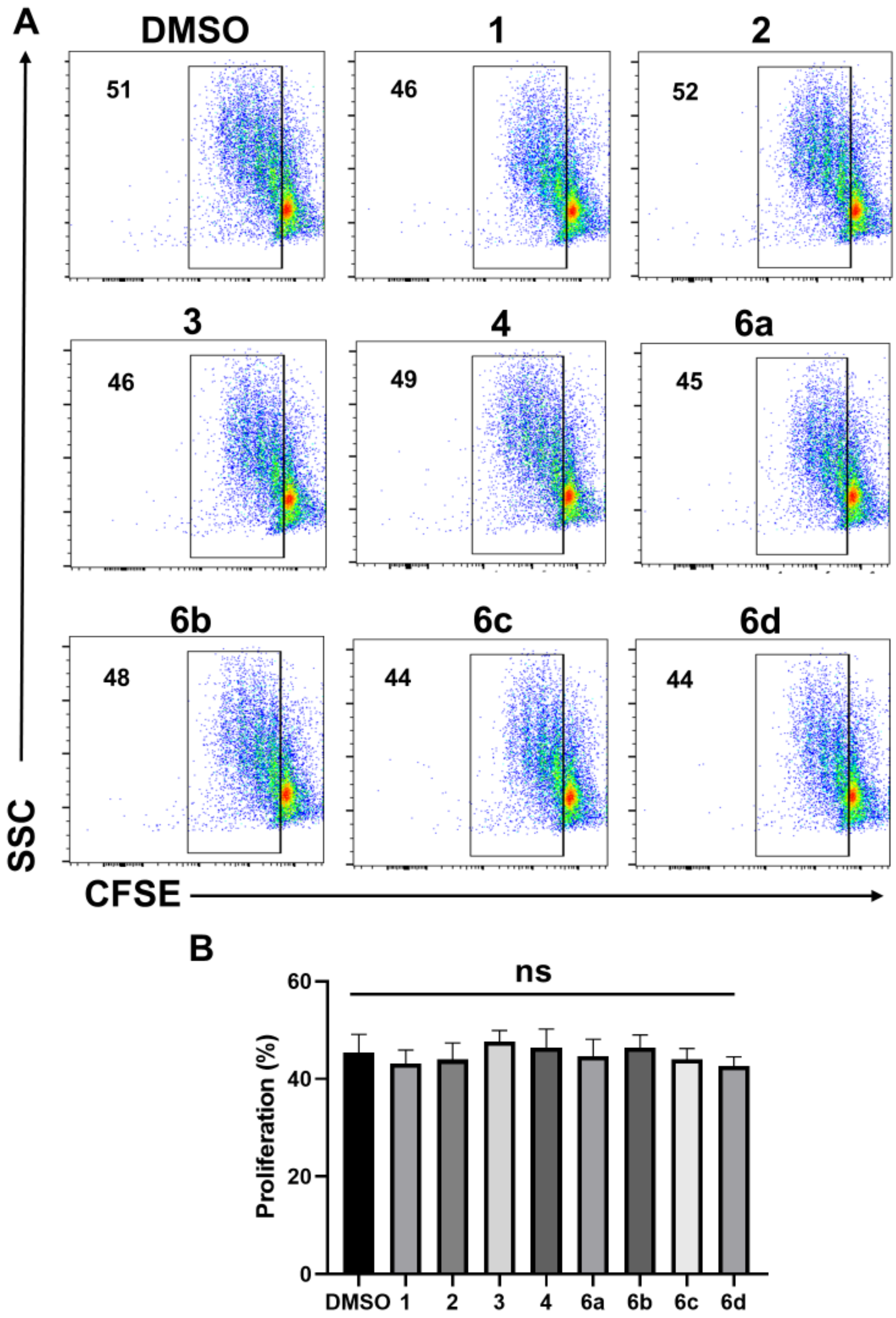

Supplementary Figure S3. The effect of *β*-AKBA and 1*H*-1,2,3-triazole derivatives at 12.5 μM concentration on T cell proliferation was determined by CFSE loss. Representative flow cytometric plots are shown in (**A**), and overall cell proliferation bar plots for all experiments performed (n = 8) are shown in (**B**).

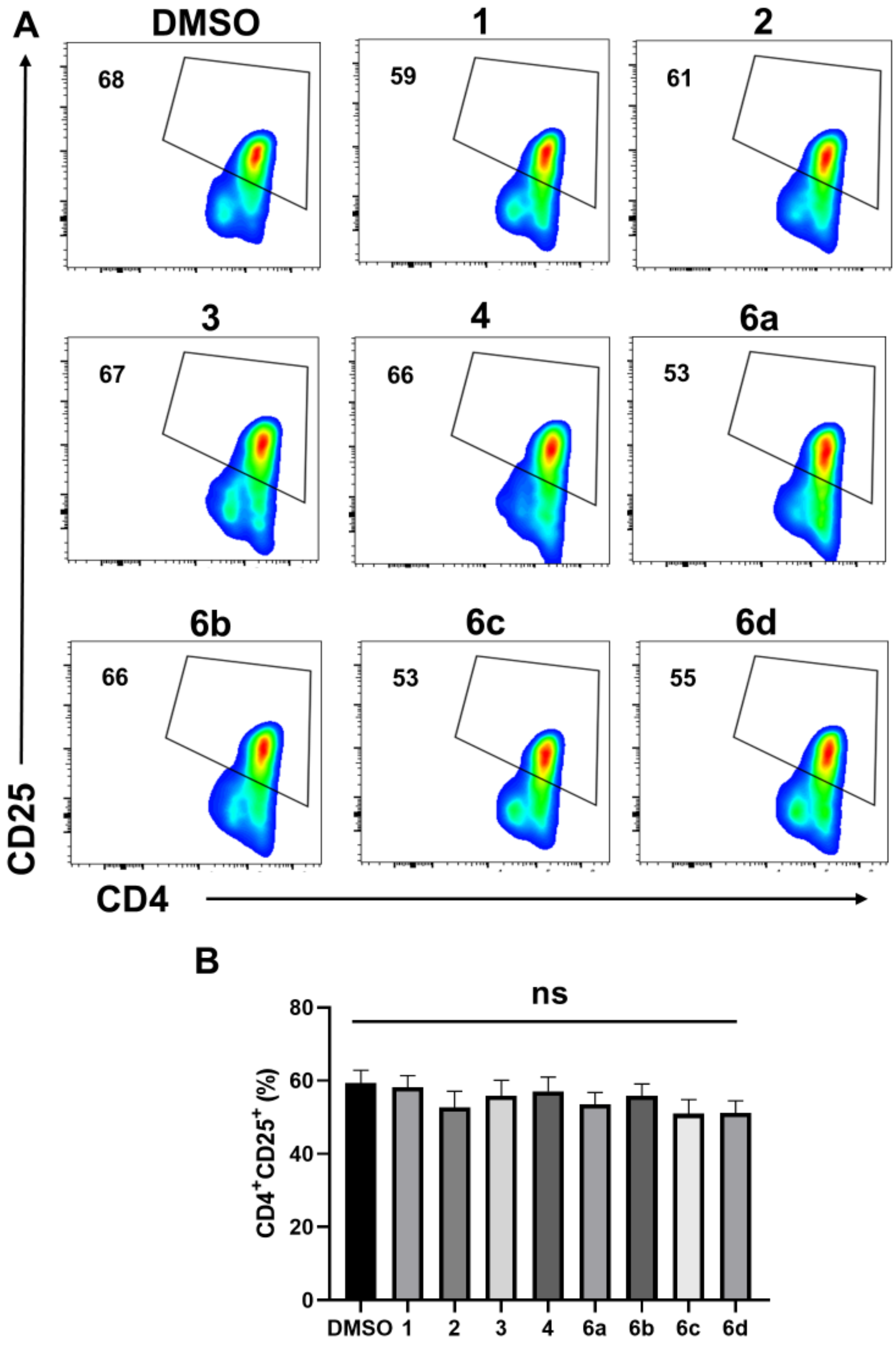

Supplementary Figure S4. Effect of β-AKBA and their derivatives at 12.5 µM concentration on expression of CD25 in CD4$^+$ T cells. Activated T cells were stained with monoclonal antibodies and analyzed by flow cytometry. Representative flow cytometric plots show gating and percentage of CD4$^+$CD25$^+$ T cells in the presence of β-AKBA and derivative compounds (**A**). The overall percentages of CD4$^+$CD25$^+$ T cells bar plots for all experiments performed (n = 8) are shown in (**B**).

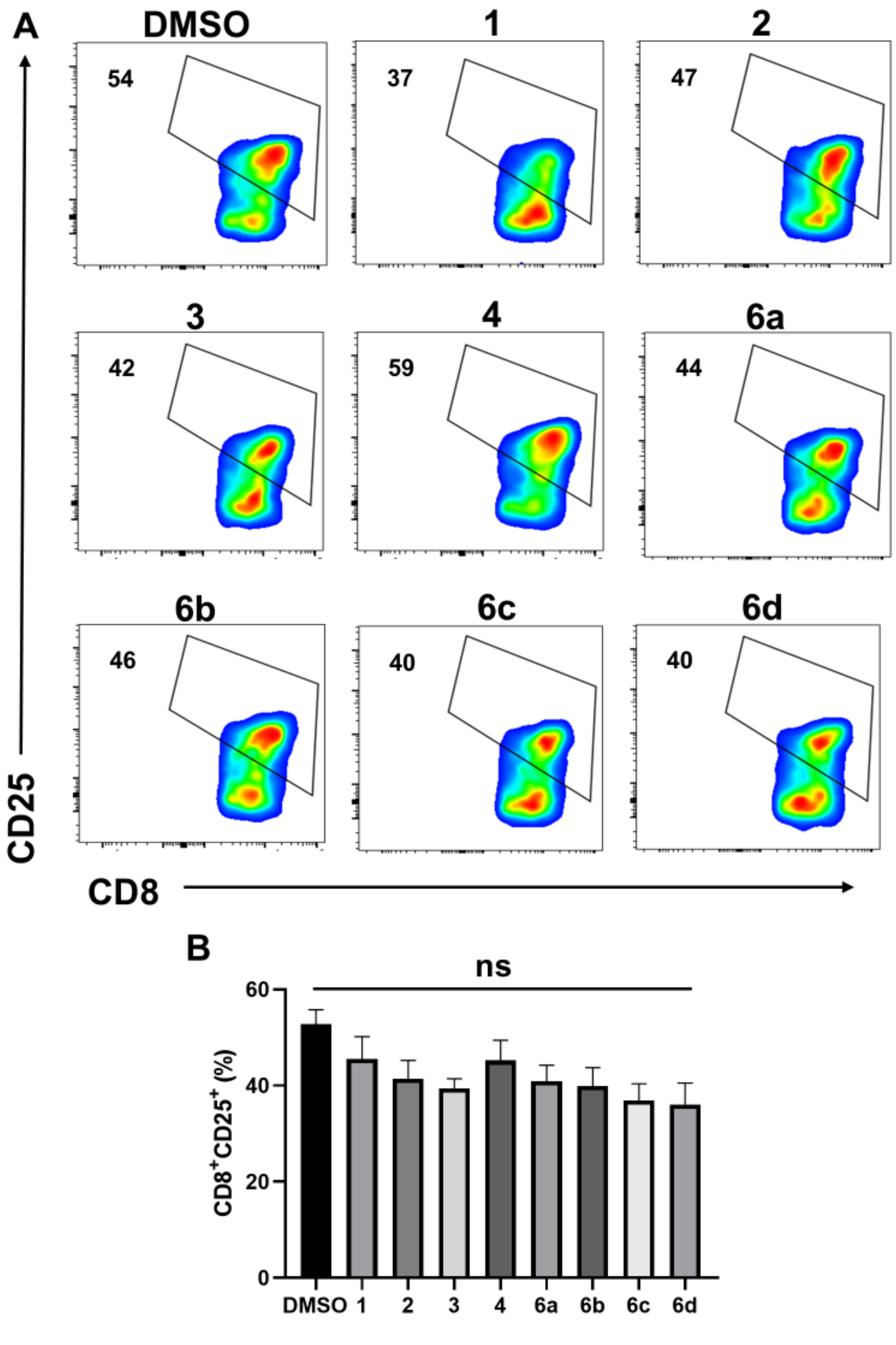

Supplementary Figure S5. Effect of *β*-AKBA and their derivatives at 12.5 μM concentration on expression of $CD25^+$ in $CD8^+$ T cells. Activated T cells were stained with monoclonal antibodies and analyzed by flow cytometry. Representative flow cytometric plots show gating and percentage of $CD8^+$ $CD25^+$ T cells in the presence of *β*-AKBA and derivative compounds (**A**). The overall percentages of $CD8^+CD25^+$ T cells bar plots for all experiments performed (n = 8) are shown in (**B**).

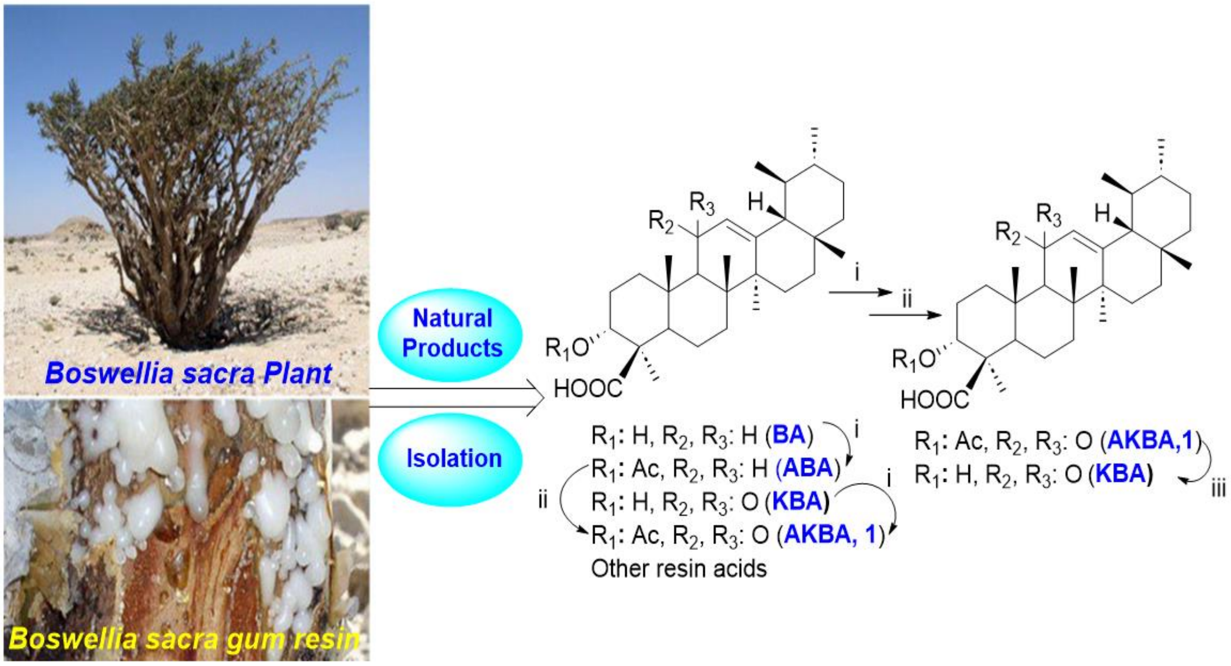

Supplementary Figure S6. Gram-scale synthesis of boswellic acids (BAs, β-AKBA, 1) from Boswellia sacra resin (BSR). Reagents and conditions: (**i**) Ac2O/Py/DMAP, CH2Cl2, RT, 6 h; (**ii**) NBS/CaCO3/H2O/hν, dioxane, RT, 10h; (**iii**) 05 N KOH in iPrOH, reflux, 10h.
